# Super-resolution visualization and modeling of human chromosomal regions reveals cohesin-dependent loop structures

**DOI:** 10.1186/s13059-021-02343-w

**Published:** 2021-05-11

**Authors:** Xian Hao, Jyotsana J. Parmar, Benoît Lelandais, Andrey Aristov, Wei Ouyang, Christian Weber, Christophe Zimmer

**Affiliations:** 1 Institut Pasteur, Imaging and Modeling Unit, UMR 3691, CNRS, Paris, France; 2 School of Public Health & Jiangxi Provincial Key Laboratory of Preventive Medicine, Nanchang University, Nanchang, 330006 China; 3 Simons Center for the Study of Living Machines, National Center for Biological Sciences (TIFR), Bangalore, Karnataka 560065 India; 4 Université de Paris, F-75013 Paris, France

**Keywords:** Chromatin, Chromosomes, Cohesin, Super-resolution microscopy, Polymer models

## Abstract

**Background:**

The 3D organization of the chromatin fiber in cell nuclei plays a key role in the regulation of gene expression. Genome-wide techniques to score DNA-DNA contacts, such as Hi-C, reveal the partitioning of chromosomes into epigenetically defined active and repressed compartments and smaller “topologically associated” domains. These domains are often associated with chromatin loops, which largely disappear upon removal of cohesin. Because most Hi-C implementations average contact frequencies over millions of cells and do not provide direct spatial information, it remains unclear whether and how frequently chromatin domains and loops exist in single cells.

**Results:**

We combine 3D single-molecule localization microscopy with a low-cost fluorescence labeling strategy that does not denature the DNA, to visualize large portions of single human chromosomes in situ at high resolution. In parallel, we develop multi-scale, whole nucleus polymer simulations, that predict chromatin structures at scales ranging from 5 kb up to entire chromosomes. We image chromosomes in G1 and M phase and examine the effect of cohesin on interphase chromatin structure. Depletion of cohesin leads to increased prevalence of loose chromatin stretches, increased gyration radii, and reduced smoothness of imaged chromatin regions. By comparison to model predictions, we estimate that 6–25 or more purely cohesin-dependent chromatin loops coexist per megabase of DNA in single cells, suggesting that the vast majority of the genome is enclosed in loops.

**Conclusion:**

Our results provide new constraints on chromatin structure and showcase an affordable non-invasive approach to study genome organization in single cells.

**Supplementary information:**

The online version contains supplementary material available at 10.1186/s13059-021-02343-w.

## Background

Major advances in understanding chromatin architecture have been made in recent years thanks to Hi-C [1] and other sequencing-based techniques that quantitatively map DNA-DNA proximity across the genome [2]. These studies have first revealed a compartmentalization of genomes into megabase-scale compartments (named A and B) that preferentially interact with other compartments of similar epigenetic signatures [1]. Later work has identified smaller, sub-megabase scale “topologically associated domains” (TADs) within which contacts are enriched [3, 4]. These domains are often associated with chromatin loops and are believed to facilitate or prevent promoter-enhancer interactions, thereby influencing gene regulation [5, 6]. The cohesin complex has been identified as a major molecular determinant of loops and TADs, since depletion of its subunit RAD21 or its chromatin loader NIPBL leads to near-complete loss of these Hi-C features in various human cell types and across species [7–10]. Modeling studies have proposed DNA extrusion mediated by cohesin as the mechanism by which loops and TADs are formed, while the insulator CTCF is believed to act as an obstacle to loop extrusion and hence helps define TAD boundaries [2, 11, 12]. Nevertheless, it remains unclear if, in what spatial size and form, and how frequently these chromatin loops and domains exist in single cells. Hi-C cannot directly address these questions because the link between contact frequencies and spatial coordinates is complex and because most Hi-C contact maps are averages over millions of cells [13–16]. Single-cell Hi-C currently delivers contact maps that are typically too sparse to reliably identify TADs or loops [17, 18]. By contrast, microscopy methods provide 3D views of chromosomes in single cells and have long been used to characterize chromosome karyotypes in metaphase spreads, where individual chromosomes are readily recognizable.

Characterizing the structure of chromosomes in interphase is more complex, because chromatin is decondensed and chromosomes no longer appear as distinct entities under conventional microscopes. Nevertheless, notable efforts have been made using fluorescence or electron microscopy to analyze interphase chromatin structure in single cells. For example, 2D super-resolution imaging of histones and 3D electron tomography combined with DNA staining have enabled to visualize heterogeneous arrangements of nucleosomes in human fibroblasts and mouse embryonic stem cells [19] and to compare chromatin fiber structure in mitosis and interphase [20]. Live cell microscopy has also been used to investigate the dynamics of chromatin in interphase, from the scale of chromosomes, chromatin domains, and single nucleosomes and its dependence on cohesin and other factors [21–24]. Fluorescence in situ hybridization (DNA-FISH) allows specific visualization of individual chromosomes or chromatin domains [25] and in combination with sequential labeling has been used to visualize many tens of genomic sites in single cells of the fly, mouse, and human and to trace chromatin regions in 3D [26–29]. One DNA-FISH study reported the existence of “TAD-like” structures in single cells, whose population averages matched TADs in bulk Hi-C data [30]. However, because similar structures were still visible in single cells after removal of cohesin, this report raised the possibility that other, cohesin-independent mechanisms, underlay TAD formation.

Here, we combine optimized generic labeling of DNA with EdU and 3D single-molecule microscopy to visualize individual chromosomes in isolation from each other in intact nuclei [31–35], and use it to analyze chromatin structure and its dependence on cohesin at high resolution in single human cells.

## Results

### Dilution labeling of individual chromosome regions

**Fig. 1:**
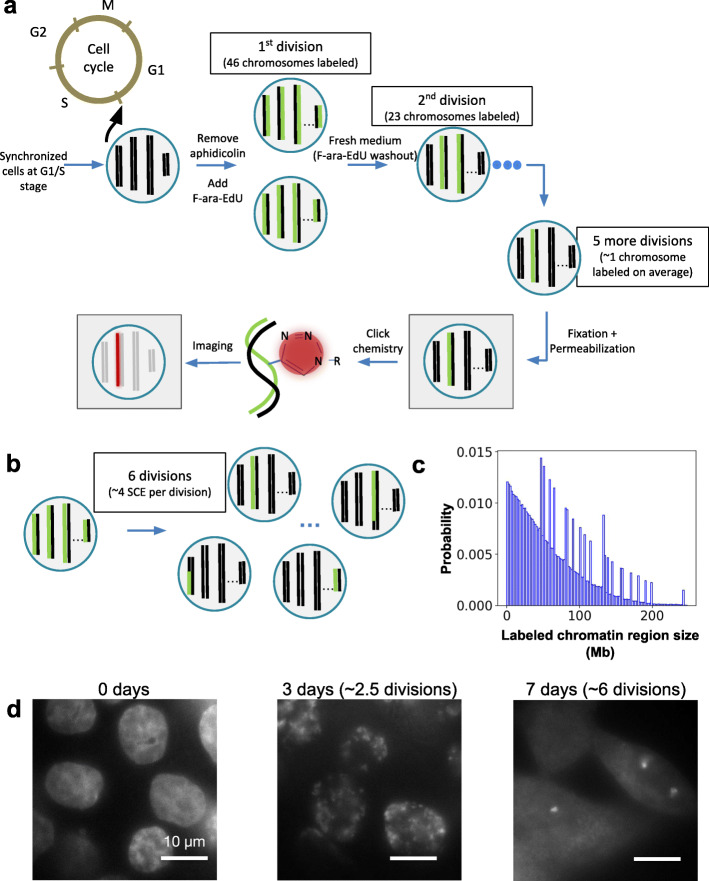
Visualizing isolated chromosomes in intact nuclei with dilution labeling. **a** Schematic of the “dilution labeling” strategy to visualize individual chromosomes. Human HCT-116 cells arrested at the G1/S transition (top left) are exposed to EdU during a single round of DNA replication, allowing EdU incorporation in newly synthesized DNA strands (green lines). As a result, each of the 46 chromosomes carries a single DNA strand with EdU modified nucleotides (1st division). Cells are then allowed to undergo multiple replication cycles in absence of EdU (2nd, 3rd,... divisions). Owing to semi-conservative replication of the DNA double helix, the number of chromosomes comprising an EdU-carrying strand is halved, on average, after each division, but EdU-carrying strands are expected to remain intact (in absence of sister chromatid exchange, see **b** below). After *m* cell divisions, cells are expected to contain only one entire EdU-carrying chromosome with probability *p*_1_ = 46 × 2^−*m*^(1 − 2^−*m*^)^45^ and no EdU-carrying chromosomes with probability *p*_0_ = (1 − 2^−*m*^)^46^, and on average <*n* >  = 46 × 2^−*m*^ labeled chromosomes (bottom right). Click chemistry with Alexa 647 dyes (red) is used to fluorescently label EdU-carrying strands after cell fixation, enabling imaging (bottom left). **b** Sister chromatid exchange (SCE) can compromise the integrity of EdU-carrying strands and lead to incompletely labeled chromosomes. **c** Predicted size distribution of EdU-labeled chromatin regions after six divisions, taking into account the measured rate of SCE (Additional file 1: Fig. S2). **d** Widefield images of EdU and A647-labeled chromosomes taken immediately after EdU incubation (0 days), after 3 days and after 7 days. As the delay between EdU incorporation and click chemistry is increased, the fluorescent signal becomes confined to a small number of micrometer-sized patches, as expected for single chromosomes

In order to directly visualize DNA, we adopted a fluorescent labeling strategy based on EdU, a chemically modified analog of thymidine that is incorporated into DNA during replication [31, 34, 36, 37]. Because EdU can cause cellular arrest and inhibit replication, we used the derivative F-ara-EdU, which has much lower toxicity (hereafter still referred to as EdU for simplicity) [32]. We synchronized HCT-116 cells in culture at the G1/S transition with a double aphidicolin block, then released the cells from the block and incubated them with EdU for 16 h, then washed EdU out. Immediately after wash-out, or after several rounds of cell divisions, we fixed and permeabilized the cells, and used click chemistry to fluorescently label EdU, before acquiring 3D widefield image stacks (Fig. 1a and “Methods”). When labeling immediately after EdU wash-out, the entire nucleus of interphase cells exhibited a roughly homogeneous staining (Fig. 1d), consistent with a uniform labeling of all 46 interphase chromosomes, and preventing the identification of individual chromosomes. However, when labeling is performed after *m* cell divisions, the semi-conservative nature of DNA replication implicates that only a subset of the chromosomes (on average, <*n* >  = 46 × 2^−*m*^) is fluorescently labeled [23, 33, 38, 39]. Indeed, images obtained after 3 days (between *m* = 2 and *m* = 3 divisions) displayed a highly inhomogeneous nuclear fluorescence, consisting of mostly isolated bright ~ 2–5 μm large clumps (Fig. 1d). These images are consistent with a minority of chromosomes being labeled and occupying distinct territories in the nucleus, as previously shown by fluorescence in situ hybridization (DNA-FISH) [25]. In images taken after 7 days (*m* ≈ 6 divisions), when the average number of labeled chromosomes is less than one (<*n* >  ≈ 0.72), cells displayed either none, one, or two bright fluorescent regions, as predicted (Fig. 1d).

To verify if our approach enables complete labeling of single chromosomes, we performed metaphase spreads with dual-labeling of EdU and DAPI [40]*.* These experiments allowed us to detect and quantify incompletely labeled chromatids as a result of recombination events, most likely sister chromatid exchange (SCE) [41] (Additional file 1: Fig. S1a). The measured recombination frequency increased with EdU concentration (Additional file 1: Fig. S1b), consistent with earlier reports showing an increase of SCE frequency with BrdU concentration [42–44]. Extrapolating our data to an EdU concentration of zero yields an estimate of 14.4% SCE per chromosome, on average (Additional file 1: Fig. S1b), in good agreement with previous estimates of spontaneous SCE in HCT-116 cells [45]. Hereafter, we use a concentration of 10 μM, for which ~ 18% of chromosomes had undergone recombination, i.e., only ~ 25% more than spontaneous levels, suggesting that EdU labeling only marginally increases SCE in our experiments. This recombination frequency implies that after 6 cell divisions, 1 − (1 − 0.2)^6^ = 74% of chromosomes will have undergone at least one SCE event (Fig. 1b). Simulations based on this SCE rate predict that the EdU-labeled chromosome regions have a wide size distribution with a median size of ~ 50 Mb, comparable to the size of chromosome 22 (Fig. 1c, Additional file 1: Fig. S2b,d). Therefore, although our method does not ensure complete labeling of entire chromosomes, it labels chromosome-scale, random, chromatin regions in a manner that enables their visual isolation from other chromosomes.

### Super-resolution 3D visualization of single chromosome regions

**Fig. 2:**
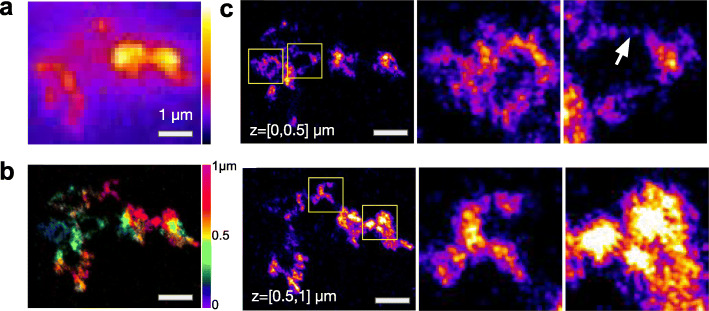
Super-resolution 3D imaging of chromosome regions. **a** Widefield 3D image of a single fluorescently labeled chromatin region (maximum intensity projection of a z-stack). **b,c** Super-resolution 3D images of the same structure obtained with ZOLA-3D [35]. **b** A color-coded view, where color indicates axial coordinate. **c** Two 500-nm-thick slices through the 3D super-resolution image are shown, with two 1 μm × 1 μm regions (yellow boxes) shown magnified to the right. The white arrow points to an isolated piece of chromatin fiber. For an animated 3D view, see Additional file 2: Video S1

The spatial resolution of widefield microscopy (> 200–300 nm laterally and > 500 nm axially) severely restricts our ability to observe internal chromosome structures such as TADs, whose sizes have previously been estimated to ~ 100–300 nm [29, 46]. We therefore turned to a single-molecule localization microscopy approach (ZOLA-3D) that uses a deformable mirror and specifically designed algorithms to reconstruct 3D super-resolution images over an axial range of several micrometers [35] (Additional file 1: Fig. S3). Our images covered an axial range of ~ 3 μm, sufficient to capture the vast majority of EdU-labeled chromatin regions and had estimated localization precisions of ~ 15–30 nm, theoretically enabling resolutions of ~ 35–70 nm (Fig. S4a-d). However, besides localization precision, SMLM resolution is also affected by the sampling of molecular positions, which is limited by EdU incorporation. A more conservative resolution criterion that takes into account sampling, Fourier ring correlation [47], suggests that the resolution of our images was in fact ~ 150 nm (Fig. S4e), significantly below the diffraction limit of ~ 325 nm given our imaging system. Indeed, these images revealed structural details that were obscured in the widefield images (Fig. 2a-c, Additional file 1: Fig. S5a, Additional file 2: Video S1). Specifically, chromosomes appeared as relatively continuous structures exhibiting highly variable, random-walk like shapes, and strong variations in EdU signal density, leading to an often granular appearance. The spatial resolution was not sufficient to trace the DNA fiber throughout chromosomes. However, some images displayed thin filaments in between more compact chromatin regions, with an apparent width of ~ 50 nm, close to the theoretical resolution predicted from localization precision, which likely correspond to single chromatin fiber stretches (e.g., Fig. 2c, arrow).

### Super-resolution 3D visualization of mitotic and interphase chromatin

We first asked if our method can reveal major structural rearrangements of chromosomes, such as those that occur during mitosis [48] and therefore imaged chromosomes arrested in G1 and M phase (Additional file 1: Fig. S5a,b, Additional file 3: Video S2). In order to quantify the physical size of the imaged chromosome regions, we first segmented the images using 3D Voronoi tessellation [49, 50], then computed gyration radii *R*_*g*_ from the segmented single-molecule localization data (“Methods”, Additional file 1: Fig. S6a,b). As expected, chromosomes were smaller in M phase than in G1, with a median gyration radius of *R*_*g*_ = 0.58 μm vs. 0.94 μm, respectively (*p* < 10^− 6^; *n* = 27–34) (Additional file 1: Fig. S5d, S7). Gyration radii are expected to strongly correlate with the genomic sizes of the imaged chromatin regions, which as mentioned above are random and highly variable (Additional file 1: Fig. S2). Indeed, gyration radii correlate strongly with the number of localizations, which is expected to increase with chromatin region size (Additional file 1: Fig. S5f). We therefore sought to characterize the shape of chromosome structures with a quantity more robust to variations in genomic size. For this purpose, we calculated Delaunay diagrams from the segmented images and computed a dimensionless “smoothness” parameter *S* based on the concave hulls of the segmented volume at spatial scales between ~ 200 nm and 1 μm laterally (~ 400 nm and 2 μm axially) (“Methods”, Additional file 1: Fig. S6c,d). This parameter falls between 0 and 100%, with higher values indicating smoother shapes. Convex shapes have *S* = 100%, and *S* is expected to be sensitive to subtle changes in the shapes of chromosomes in this range of scales (~200 nm - 2 μm), such as the presence or absence of holes. Unlike gyration radii, the measured smoothness values did not correlate significantly with localization counts (Additional file 1: Fig. S5g), suggesting robustness to chromatin region size. We measured an increase in median smoothness for M phase chromosomes (*S* = 75.4%) compared to G1 phase (*S* = 69.2%), as expected (*p* = 1.5 × 10^− 3^; *n* = 27–34) (Additional file 1: Fig. S5e, S7). In addition, chromosomes in M phase appeared more homogeneous and mostly lacked the substructures apparent in the G1 chromosomes (Additional file 1: Fig. S5a,b). This observation is consistent with the disappearance of chromatin domains previously observed by Hi-C in mitotic chromosomes [48, 51]. These results illustrate our imaging technique’s ability to assess changes in chromosome structure visually and quantitatively.

### Effect of cohesin depletion on high-resolution chromatin structures

**Fig. 3:**
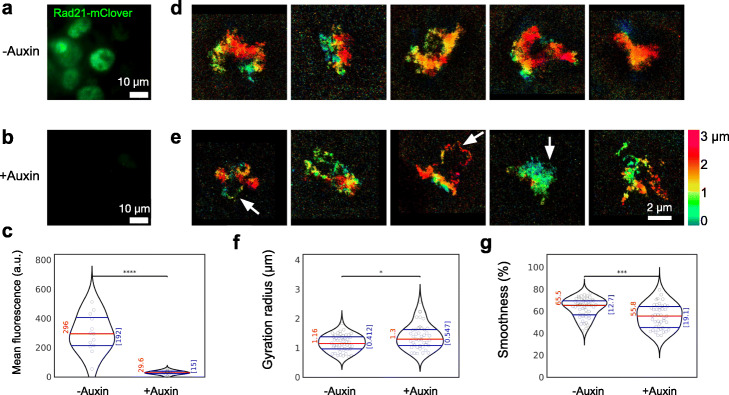
Visualizing and quantifying cohesin-dependent chromatin structures. **a,b** Auxin-dependent degradation of cohesin. HCT-116-RAD21-mAC cells [52], in which the cohesin subunit RAD21 is auxin-degradable and fused to the fluorescent protein gene mClover, were treated with 500 μM of auxin for 6 h (**a**) or left untreated (**b**). **c** Comparison of mean fluorescence in auxin-treated vs untreated cells confirms that auxin leads to efficient degradation of RAD21-mClover (*p* < 10^− 5^). **d,e** Example 3D super-resolution images of individual chromosome regions in HCT-116-RAD21-mAC cells left untreated (**d**) or treated with auxin (**e**). The arrows in **e** point at isolated stretches that likely correspond to single chromatin fibers, with approximate lengths of 1.6 μm, 3.2 μm, and 0.5 μm, from left to right. Color indicates axial coordinates. For animated 3D views, see Additional file 4: Video S3. **f,g** Violin plots compare the distributions of gyration radii (**f**) and smoothness (**g**) in cells with (*n* = 43) or without (*n* = 50) cohesin depletion by auxin treatment. Median gyration radii differ significantly (*p* = 0.013), as do smoothness values (*p* = 5 × 10^− 4^) (two-sided rank sum tests). See also cumulative distribution functions and bootstrap analyses in Additional file 1: Fig. S7 and S8d

We next aimed to analyze the much more subtle effect of cohesin on interphase chromatin structures. For this purpose, we turned to a modified HCT-116 cell line, where the cohesin subunit RAD21 is fused to a degron allowing to induce auxin-dependent degradation of the protein. In addition, the protein is labeled with the fluorescent protein mClover to enable its visualization [8, 52]. Effective depletion of RAD21 upon auxin treatment was confirmed by a strong reduction of nuclear RAD21-mClover fluorescence (Fig. 3a–c). Super-resolution ZOLA-3D images of these cells again exhibited large cell-to-cell variability, both in absence and in presence of auxin (Fig. 3d,e, Additional file 4: Video S3). Nonetheless, cohesin depletion led to a 12% increase in chromosome size from a median *R*_*g*_= 1.16 μm to *R*_*g*_ = 1.30 μm (*n* = 43–50; *p* = 0.013) and a 15% decrease in median smoothness from *S =* 65.5 % to *S* = 55.8% (*n* = 43–50; *p* = 5 × 10^− 4^) (Fig. 3f,g, Additional file 1: Fig. S7). Although these changes were relatively small, bootstrap analyses confirmed that these differences are significant and not an artifact of sampling a highly variable structure population (Additional file 1: Fig. S8d-f).

We note that the gyration radii in absence of auxin were already slightly larger than in the wild-type HCT-116 cell line (*p* = 0.002) and that the smoothness was smaller (*p* = 0.048) (Additional file 1: Fig. S8a,b). A bootstrap analysis and statistical comparisons of the entire distributions confirmed the statistical significance of the difference in gyration radii, but not in smoothness, suggesting that chromosome shapes, despite their large cell-to-cell variability, were not significantly altered in the genetically engineered cell line (Additional file 1: Fig. S8c, S7).

### Multi-scale polymer model of human chromosomes

**Fig. 4:**
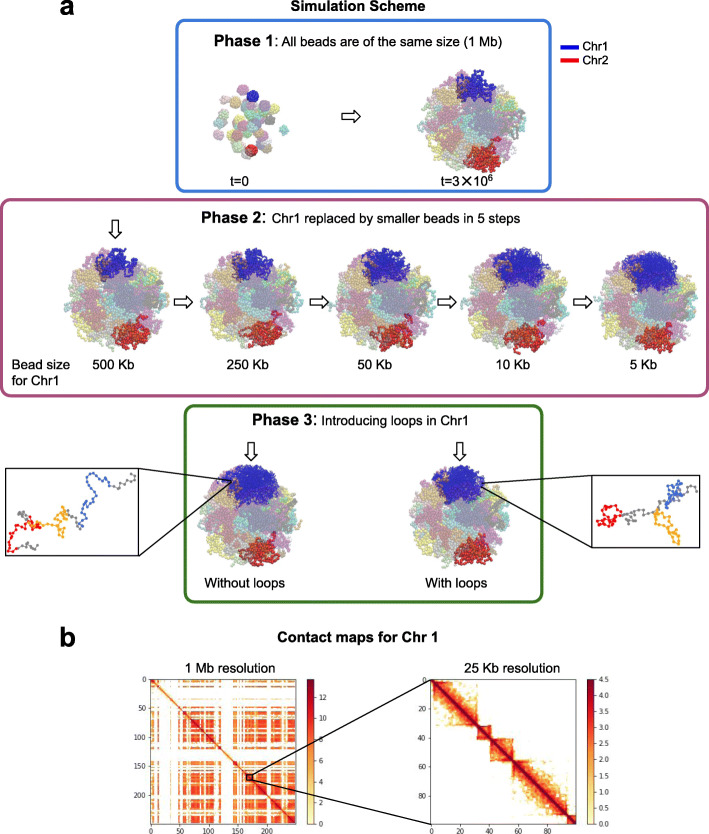
Multi-scale whole nucleus simulations of chromosomes with compartments and loops. **a** Multi-scale whole nucleus simulation approach. Shown are simulation snapshots, where each of the 46 chromosomes is modeled as a polymer chain with a distinct color. Chromosome 1 is highlighted in blue, chromosome 2 in red. Simulations proceed in three consecutive phases. In phase 1, all chromosome chains are coarse grained, with 1 bead for every megabase of DNA, and *t* = 3 × 10^6^ iterations of Langevin dynamics are computed. In phase 2, chromosome 1 is fine-grained progressively in five successive stages. At each stage, beads of chromosome 1 are replaced by 2 or 5 smaller beads, each representing 2 or 5 times smaller DNA segments, until each bead corresponds to 5 kb of DNA. All other 45 chromosomes are kept coarse-grained (1 Mb beads), and *t* = 10^5^ iterations are computed at each stage. In phase 3, a specified number of loops can be formed by creating bonds between non-consecutive bead pairs, and *t* = 10^5^ iterations are computed. Insets to either side of the green rectangle show a polymer segment featuring 3 loops in different colors (right) or no loops (left). The simulations in all three phases also include energy potentials to model the formation of A/B compartments. See “Methods” for details. **b** Contact frequency matrices for chromosome 1 as predicted by a simulation with compartments and 15 loops/Mb (logarithmic scale). Left: entire chromosome 1 with bins of 1 Mb. Alternating A/B compartments are clearly visible. Right: a 2.5-Mb region on chromosome 1 with bins of 25 kb. Blocks of enriched contact frequencies consistent with TADs are clearly seen along the diagonal. Color bar indicates the natural logarithm of counts per bin

We then asked what changes in chromatin structure might be expected in the super-resolution images if cohesin depletion leads to disruption of chromatin loops in single cells. To address this, we turned to in silico models of chromosomes in the nucleus based on polymer physics [46, 53]. Specifically, we implemented a multi-scale simulation approach that allowed us to model one chromosome at high genomic resolution (5 kb), while still accounting for the constraints exerted by the 45 remaining chromosomes in the nucleus, which we modeled at 1 Mb resolution to keep computations tractable (Fig. 4). This approach enables us to capture the micrometer-scale shapes of large (e.g., 50 Mb) chromosome regions, the Mb-scale organization of epigenetic (A/B) compartments [1], and finer structures such as 50–500-kb large chromatin loops. Following earlier work [46], we modeled A/B compartments by means of attractive interactions (between B compartments), which led to predicted contact correlation matrices in relatively good agreement with Hi-C data [8] (Additional file 1: Fig. S9). More importantly for this study, our simulations also allow for the presence of a variable number of chromatin loops. We created these loops at random locations biased by CTCF sites, consistent with their assumed role as obstacles to loop extrusion [11, 12] (“Methods”, Additional file 1: Fig. S10a,b). Because the frequency of loops in single cells is poorly constrained by the Hi-C data, we allowed the number of simulated loops per cell to vary from 0 to 25 per Mb. Our model predicted contact frequency maps with enriched contact domains along the diagonal and contact frequency peaks with a genomic size distribution similar to the Hi-C data [8, 46] (“Methods”, Fig. 4b, Additional file 1: Fig. S10c).

**Fig. 5:**
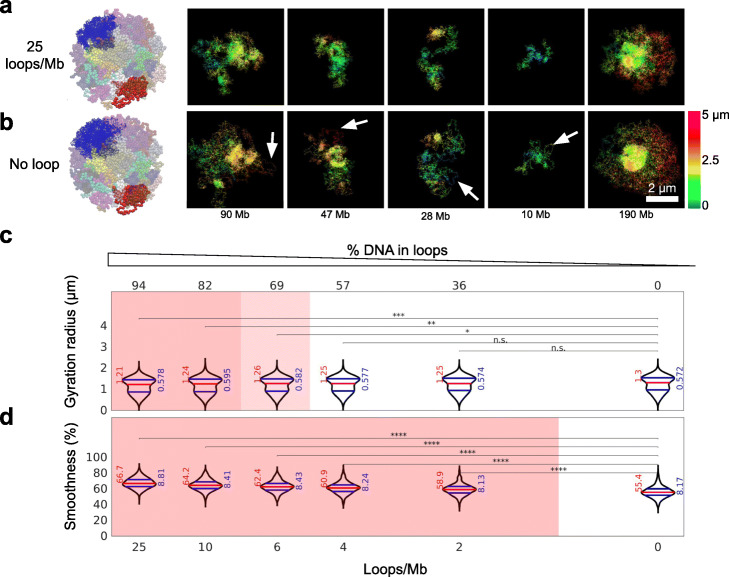
Modeling and quantifying the effect of cohesin-dependent loops on chromatin structure in silico. **a,b** Chromosome structures and images generated by our multi-scale polymer simulation. The five images show example 3D super-resolution views of simulated chromosome regions, with (**a**) or without (**b**) loops. The genomic size of each region is indicated and ranges from 10 to 190 Mb. A snapshot of the simulated model is shown to the left. For animated 3D views, see Additional file 5: Video S4. **c**. Violin plots show the distribution of gyration radii and smoothness as function of loop counts per Mb or, equivalently, percentage of DNA enclosed by loops. Pink shading indicates where median values differ with intermediate significance (*p* < 0.05, light shade), or with high significance (*p* < 0.01, darker shade) from values in absence of loops (two-sided rank sum tests)

In order to compare modeled 3D chromatin structures with our imaging data, we first simulated the entire 249-Mb-long chromosome 1, then extracted random chromatin regions with a genomic size distribution as predicted above to mimic the variability resulting from random chromosome labeling and SCE (Additional file 1: Fig. S2c,d, S11a). From these extracted polymer chains, we then simulated 3D super-resolution images using localization counts similar to the experimental data, introducing similar levels of background noise and random localization errors that lead to FRC resolutions similar to the experimental data (“Methods”, Additional file 1: Fig. S11a,b, S4e). The resulting images exhibited highly variable shapes, as expected, characterized by inhomogeneous densities, frequent presence of high-density regions, and occasional isolated stretches of polymer (Fig. 5a,b, Additional file 1: Fig. S11c, Additional file 5: Video S4). Similar features were also observable in our experimental data in G1 phase above (Figs. 2, 3). The high-density regions were clearly caused by A/B compartmentalization, since removal of the attractive interactions between B compartments led to their disappearance (Additional file 1: Fig. S12).

### Comparison of predicted and observed 3D chromatin structures

Further inspection of these simulated images for different numbers of loops suggested that removal of loops leads to the increased appearance of large stretches of polymers in relative isolation from the bulk mass of the polymer (“loose stretches”) (Fig. 5b, arrows; Additional file 1: Fig. S11c). Visualization of our experimental data also revealed large (up to ~ 3 μm long) loose stretches in cells depleted of cohesin (Fig. 3e, arrows).

To characterize structural similarities or differences quantitatively, we computed gyration radii and smoothness of the simulated images, as done above for the experimental data and analyzed their behavior for different numbers of loops (Fig. 5c,d). Because of the variability in polymer shapes, compounded by the variability in chromatin region size, predicted gyration radii and smoothness varied substantially between configurations (Fig. 5c,d), much as between cells in experimental data. Despite this variability, median gyration radii and smoothness changed significantly when increasing the number of chromatin loops from 0 to 25 loops/Mb (on average), with more loops leading to smaller gyration radii and larger smoothness (Fig. 5c,d, Additional file 1: Fig. S7). The predicted smoothness depended sensitively on loop number and already increased significantly (*p* < 10^− 10^) when adding 2 loops/Mb (Fig. 5d, Additional file 1: Fig. S7).

Importantly, this predicted behavior is in accordance with the above-reported experimental data (smaller gyration radii and larger smoothness in untreated cells vs. auxin-treated cells, see Fig. 3f,g), assuming that cohesin depletion leads to loss of chromatin loops in single cells. Beyond this qualitative consistency, we note that our predictions are in surprisingly good quantitative agreement with the experimental data (Figs. 3f,g, 5c,d). Both the predicted median gyration radius and smoothness in absence of loops (*R*_*g*_= 1.30 μm and *S* = 55.4%) are within 1% of their experimental counterparts in images of auxin-treated cells (*R*_*g*_= 1.30 μm and *S* = 55.8%). Furthermore, our predictions for simulations with 25 loops/Mb (median *R*_*g*_= 1.21 μm and *S* = 66.7% respectively) are also very close (within 1–4%) to the experimental values for untreated cells (median *R*_*g*_= 1.16 μm and *S* = 65.5%, respectively). For simulations with fewer loops, the agreement with the data was less good. For example, with 4 loops/Mb, the predicted gyration radii did not significantly differ from simulations without loops, and the smoothness increased less than in experimental data of untreated vs cohesin-depleted cells (9.9% vs 17.4%). Based on these comparisons, we estimate that the number of loops present simultaneously in single cells is at least ~ 6–25 per Mb. This estimate implies that 70–94% or more of the nuclear DNA is inside at least one loop in single interphase cells. Thus, our data and analysis support the notion that the vast majority of the genome in each cell is enclosed in loops.

## Discussion

The above estimate of loop density suggests a total number of concurrent loops in the 6.4 Gb HCT-116 genome of ~ 38,000–160,000 or more. Since our estimates are based on a comparison between imaging data and model predictions, we acknowledge that they are contingent on a number of assumed parameters, such as simulation time, loop extrusion processivity, or single-molecule localization errors. Although these parameters were chosen based on orthogonal data (e.g., Hi-C data and resolution estimates), they are imperfectly known, and changing some of these parameters can affect the agreement between predictions and imaging data and hence our estimate of loop density (Additional file 1: Fig. S13). It is therefore instructive to confront these numbers to independent evidence. Recent imaging studies have used fluorescence correlation spectroscopy, fluorescence recovery after photobleaching, and single-molecule tracking to estimate the number of bound or freely diffusing cohesin molecules in mouse embryonic stem cells and HeLa cells [54, 55]. This led to an estimated ~ 160,000 chromatin-bound cohesin complexes in a single HeLa cell [55]. Assuming similar chromatin loop densities, and accounting for the different genome sizes (7.9 Gb for HeLa vs 6.4 Gb for HCT-116), our orthogonal estimate predicts at least ~ 47,000–200,000 simultaneous loops in the HeLa genome. These independent quantifications of loops and cohesin complexes are thus consistent with each other in the framework of cohesin-mediated DNA extrusion, whether extrusion involves a single cohesin complex or two. Thus, our study provides fresh evidence for the existence of cohesin-dependent chromatin loops as well as new and surprisingly high estimate of their frequency in single cells. We also note that this estimate is comparable to the roughly twenty consecutive loops per megabase that were proposed to form through the action of condensin II at the beginning of mitotic chromosome condensation in a Hi-C study of the chicken genome [51].

A recent study using multiplexed DNA-FISH reported the existence of “TAD-like” structures in single HCT-116 cells, with population averages matching TADs in bulk Hi-C data [30]. However, because similar structures were still visible in single cells after removal of cohesin, this report raised the possibility that chromatin domains might be maintained by additional factors besides cohesin. Instead, our data are consistent with a model that postulates loops maintained by cohesin only. Note that spatial domains reminiscent of TADs emerge from our simulations even in the absence of loops (Additional file 1: Fig. S14).

At the technical level, our results highlight the complementarity of EdU-based labeling approaches with DNA-FISH to study 3D chromatin architecture [56]. DNA-FISH allows sequence-specific visualization of individual chromosomes, chromatin domains, or sets of chromatin loci [25–29, 57]. Despite their power, DNA-FISH methods suffer limitations. First, DNA-FISH requires melting of the DNA double helix and is therefore susceptible of altering chromatin structures, especially at small (e.g., subdiffraction) scales [58, 59]. Second, the need for large sets of custom-designed oligonucleotide probes entails significant cost, making extensions to entire chromosomes or genomes unaffordable. Third, repeated regions remain ambiguous. By contrast, EdU labeling does not necessitate DNA denaturation, thereby better preserving chromatin structures, is highly cost-effective and can be applied to any genome without knowledge of its sequence, including repeated regions [31–33, 36, 56]. While EdU does not offer the sequence-specific information provided by FISH and stains chromosomes randomly, EdU provides a generic labeling of large (chromosome-scale) chromatin regions that is largely uniform except for regions with very low AT content (Additional file 1: Fig. S15). We note that specific DNA-FISH labeling of chromosomes using, e.g., Oligopaint probes [26], is compatible with EdU labeling, potentially allowing to combine some of the advantages of both techniques (Additional file 1: Fig. S16).

Despite these advantages, our approach is not without limitations and potential for improvements. One limitation is sister chromatid exchange (SCE). At the EdU concentrations used here, SCE is only marginally increased above spontaneous levels by the labeling; nevertheless, even spontaneous SCE events lead to the visual fragmentation of chromosomes. Therefore, it may be worth exploring strategies to reduce SCE frequency below spontaneous levels, e.g., using cell lines less prone to SCE, or depriving cells of oxygen, or by knocking out genes involved in SCE, such as TREX2 [60]. Another caveat are potential alterations of chromatin structure due to the labeling method. Although EdU labeling is less invasive than hybridization-based approaches, we cannot rule out that incorporation of modified nucleotides modifies chromatin structure locally. However, we note that F-ara-EdU, as opposed to BrdU or EdU, has little or no effect on DNA replication and does not cause cell cycle arrest at the concentrations enabling fluorescent labeling used here [32], suggesting that F-ara-EdU does not impact chromatin structure in a functionally detrimental manner. If the abovementioned issue of SCE can be remedied, our imaging approach could in principle be combined with higher F-ara-EdU concentrations to determine potential effects of the labeling on chromatin structure. Increasing F-ara-EdU concentrations could potentially also help increase the spatial resolution of our images, which appears to be limited by the small percentage of labeled and localized thymidine nucleotides rather than by single-molecule localization precision (Additional file 1: Fig. S4). If labeling efficiency can be augmented to the point where localization precision becomes the limiting factor instead, techniques that improve on the fluorescent yield or photon collection, such as dual objective microscopy [61], should enable higher resolution views and quantifications of chromatin structure.

Notwithstanding these caveats and potential for improvement, our approach thus provides an attractive complement to FISH-based techniques among the growing toolbox for studying the 4D nucleome in single cells.

## Conclusion

We described a combined experimental and computational strategy to visualize, quantitatively analyze, and simulate chromatin regions in 3D at high resolution. Our experimental results strengthen the view of the cohesin complex (and its subunit RAD21) as a crucial factor for chromatin loop maintenance in single cells and are in line with polymer simulations postulating exclusively cohesin-dependent loops. The observed chromatin loosening as a result of cohesin depletion and our quantitative estimates of loop densities constitute important new features of chromatin organization that previous approaches left unresolved.

## Methods

### Cell culture and synchronization

HCT-116 cells (*Homo sapiens* colon colorectal carcinoma) were grown in DMEM (Dulbecco’s modified Eagle’s medium) supplemented with 10% FBS and 1× penicillin/streptomycin at 37 °C, with 5% CO_2_ in a humidified incubator. HCT-116-RAD21-mAID-mClover cells (HCT-116-RAD21-mAC) were kindly provided by Prof. Kanemaki [52]. Cells were cultured in McCoy’s 5A medium supplemented with 10% FBS, 2 mM L-glutamine, and 1× penicillin/streptomycin at 37 °C with 5% CO_2_. The AID-tagged RAD21 was degraded by the addition of 500 μM auxin (indole-3-acetic acid, IAA; Sigma Aldrich) for 6 h. Cells were synchronized at the G1/S transition with a double aphidicolin block. In brief, cells were incubated with aphidicolin (1 μM) for 16 h, released from the block for 8 h, and then incubated with aphidicolin again for 16 h to block DNA synthesis. To harvest cells in G1 phase, the cells were first grown in serum-free DMEM overnight (ca. 16 h), then in fresh DMEM containing 1 μM aphidicolin for 3 h; cells were allowed to enter G1 phase, but not S phase. To harvest cells in M phase, the cells were cultured in DMEM with 100 ng/μL nocodazole and stopped in metaphase.

### Dilution labeling of single chromosomes

For chromosome labeling, we released cells from the aphidicolin block by a triple wash-out with 10 mL of PBS, exchanging with label-free medium and immediately adding (2′S)-2′-deoxy-2′-fluoro-5-ethynyluridine (F-ara-EdU) to a final concentration of 5–10 μM. We incubated cells with F-ara-EdU for ca. 16 h to cover the entire S phase and in order to label chromosomes entirely. After removal of F-ara-EdU, cells were grown in fresh DMEM for 5 days, then were transferred to glass coverslips to facilitate imaging, and grown for 2 more days. The independent segregation of F-ara-EdU-carrying and unlabeled chromosomes during each replication round effectively dilutes labeled chromosomes in the nucleus. On average, less than one single chromosome per nucleus is expected to be labeled with F-ara-EdU after 7 days (Fig. 1a).

### Cell fixation and click chemistry labeling

Cells were fixed with 4% PFA for 15 min at room temperature and permeabilized with 0.5% Triton X-100 for 20 min. After blocking with 3% BSA in PBS for 30 min at room temperature and washing with PBS, cells were incubated upside-down on 50-μL drops of freshly prepared staining mix (10 μM AlexaFluor 647 azide, 4 mM CuSO_4_, and 10 mM sodium ascorbate in PBS) for 30 min at room temperature in the dark. Cells were washed first with PBS for 2 min, then with 0.5% Triton X-100 in PBS for 2 min, and then again twice with PBS for 2 min. To image chromosomes in metaphase spreads, F-ara-EdU was added to the medium during DNA replication, and cells were incubated overnight. Nocodazole was added to the medium to arrest cells in M phase and F-ara-EdU-labeled cells were incubated at 37 °C and 5% CO_2_ for 18 h. Cells were washed with PBS, harvested with shake-off, and spinned down. After spinning down the cells, the pellet was resuspended in 10 volumes of 3:1 (v/v) fresh MeOH/AcOH and incubated at room temperature for 60 min. The cell suspension was dropped on a glass slide from a height of at least 30 cm, then cells were fixed with 4% PFA and treated with 0.5 mg/mL pepsin at room temperature for 10 min. Cells were then subjected to click chemistry labeling as above and DAPI staining.

### Super-resolution 3D imaging

Each chromosome region imaged in super-resolution belongs to a distinct cell, and images were obtained from at least three samples per condition. All super-resolution imaging experiments were carried out with ZOLA-3D, a custom-built 3D single-molecule localization microscopy (SMLM) system featuring a deformable mirror and a water immersion objective (1.2 NA) and operated using a saddle-point PSF [35]. Image acquisition was restricted to labeled chromosome regions contained entirely within the ~ 3 μm axial range of ZOLA-3D. This led to rejection of only a small number of cells, because the vast majority of labeled chromatin regions had much smaller axial than lateral extent (Additional file 1: Fig. S4f). Laser light at 640 nm was used to excite Alexa Fluor 647, and after acquisition of 20,000 frames, laser light at 405 nm was used to reactivate dyes in the triplet state. For all 3D SMLM imaging experiments, we used HILO illumination at 500 mW laser power and acquired between 50,000 and 100,000 frames, with 50 ms exposure time. Imaging was done using a photoswitching buffer consisting of 50 mM Tris-HCl (pH 8.0) with 10 mM NaCl, 10% glucose, 1% v/v 2-mercaptoethanol (Sigma), 168 AU glycose oxidase (Sigma), and 1400 AU catalase (Sigma). All images were reconstructed with the ZOLA-3D ImageJ plugin [35].

### Analysis of localization data

Simulated and experimental 3D SMLM data were analyzed using custom-written Python scripts. We implemented a 3D segmentation method based on Voronoi tessellation, which is suited for SMLM data and requires few hyper-parameters [49, 50]. For any given set of localizations (corresponding to an individual imaged or simulated chromatin region), we first computed a Voronoi tessellation, which defines 3D cells around each localization. For each localization ***x***_*i*_, we then defined a local neighborhood as the set of cells located no more than *k* cells away from the current cell. The local density at ***x***_*i*_ was then calculated as the number of localizations in the neighborhood divided by the volume of the neighborhood cells. Small *k* can lead to an overestimation of the local density due to repetitive localizations of the same molecule, whereas large *k* can lead to an underestimation of the local density of small regions [49]; we empirically set *k* to 2. Next, we performed semi-automatic thresholding to remove background localizations. For this purpose, we manually defined a region of interest in the background, computed the local density as above, and defined the threshold as 4 times the average density in this region. All localizations for which the local density fell below this threshold were eliminated and the remaining *N* localizations ***x***_*i*_ (*i* = 1. *N*) were considered as valid localizations from the imaged (or simulated) chromatin region. We measured the size of this chromatin region *R*_*g*_ by the gyration radius, defined as ${R}_g^2=\frac{1}{N}{\sum}_{i=1}^N{\left\Vert {\mathbf{x}}_i-\frac{1}{N}{\sum}_{i=j}^N{\mathbf{x}}_j\right\Vert}^2$. We further computed a 3D Delaunay triangulation of these *N* localizations, resulting in a set of tetrahedra connecting all localizations together and forming a convex hull. We then removed tetrahedra whose size, as defined by the radii of their circum-ellipsoid, is below a threshold α, which results in a 3D α-concave hull [62]. We computed two α-concave hulls, for α_1_ = (100,100,200) nm and α_2_ = (500,500,1000) nm. The α_1_-concave hull allows us to define fine structures close to the estimated resolution of our images (Additional file 1: Fig. S4), whereas the α_2_-concave hull provides a coarser structure (Additional file 1: Fig. S6c,d). The smoothness of the chromatin region was computed as $S=100\kern0ex {V}_{\alpha_1}/{V}_{\alpha_2}$, where ${V}_{\alpha_i}$ is the volume of the concave hull for α = α_*i*_. *S* is comprised between 0 and 100%, with *S* = 100% for a smooth shape, e.g., a convex shape.

We used violin plots to compare gyration radii and smoothness distributions between different conditions (e.g., Figs. 3 and 5), with blue lines indicating the first and third quartiles, the blue value indicating the interquartile range, and a red line and red value indicating the median. In some plots, the individual data points are also shown as light gray circles.

### Polymer simulations

In our simulations, chromosomes were modeled as chains of beads undergoing Langevin dynamics inside a confining nuclear sphere and subject to steric constraints [53]. We used a multi-scale modeling approach designed to simulate a single chromosome at fine genomic scales, while still modeling the presence of the other 45 chromosomes. Our simulations also account for the two main levels of chromatin organization: A/B compartments and loops/TADs. Specifically, we simulated the dynamics of chromosomes in three phases as follows (Fig. 4a). In the first phase, we coarse-grained all 46 chromosomes at a genomic resolution of 1 Mb, where each 1 Mb segment of chromatin was represented by a single bead of diameter 300.0 nm (this size was defined to obtain a final overall volume occupancy of ~ 12%). To model the formation of A/B compartments, each bead was assigned to either A or B compartments depending on the eigenvectors (first principal component of the Pearson correlation matrix) obtained from the Hi-C data [1, 8] using Juicer [63]. A small attractive Lennard-Jones potential was applied between all B-B pairs while all other bead pairs (A-A and A-B) interact via purely repulsive Lennard-Jones potentials. These 46 polymer chains were confined in a spherical volume of 4.95 μm radius and subjected to Langevin dynamics for 3✕10^6^ iterations (Fig. 4a, blue rectangle). In the second phase, we model chromosome 1 (the longest chromosome, with a size of 249 Mb) at progressively higher genomic resolution, from 1 Mb to 5 kb, using four intermediate resolutions of 500 kb, 250 kb, 50 kb, and 10 kb (Fig. 4a, pink rectangle). For each increase in genomic resolution (fine-graining), we take the chromosome configurations of the last iteration, replace each bead of chromosome 1 by the appropriate number of smaller beads, and allow the simulation to proceed for 10^5^ iterations. Repeating this procedure 5 times led to a fine-grained chain for chromosome 1, where each bead represents 5 kb and has a diameter of 45 nm. After each resolution increase, the positions of the smaller beads were obtained by random Gaussian displacement (with a standard deviation of 45 nm along each coordinate) from the center of the original (larger) beads. All other 45 chromosomes were kept coarse grained at 1 Mb resolution. The radius of the nucleus was increased progressively from 4.95 to 5.4 μm to prevent the randomly displaced smaller beads from moving out of the nucleus and the size of the beads was adjusted to maintain a volume occupancy of 12% [64]. In the third phase of the simulations, we allow for the formation of loops (Fig. 4a, green rectangle). To do this, we connected pairs of beads by spring potentials with the same interaction potential as between consecutive beads. The selection of bead pairs (loop anchors) was designed to mimic the effect of loop extrusion [11, 12] and to be consistent with genomic loop sizes determined by Hi-C. Specifically, we first defined the genomic positions of potential extrusion obstacles, e.g., CTCF binding sites (red dots in Additional file 1: Fig. S10a), by randomly sampling the probability distribution $f(L)=L/{L}_0\ {e}^{-L/{L}_0}$ where *L*_0_ = 165 kb (Additional file 1: Fig. S10b). This density leads to an average loop size of 320 kb (median 275 kb) that matches the observed distribution of cohesin-dependent loop sizes in Hi-C data for HCT-116 cells [8, 46]. Next, for each loop, we first selected a random genomic position on chromosome 1 (corresponding to the loading point of a DNA extrusion complex), assigned it to both loop anchors, and subsequently moved each anchor in either direction by genomic distances *d*_*L*_ and *d*_*R*_ drawn from the probability density $f(d)\propto {e}^{-d/{d}_0}$, where *d*_0_= 250 kb represents an average processivity. If either anchor moves beyond an obstacle (either a CTCF binding site or another previously defined bead anchor), we stop it there. We performed several sets of simulations for different numbers of loops, from 0 (no loops) up to 25 loops per megabase (on average), with intermediate simulations for 2, 4, 6, 10, 15, and 20 loops/Mb, as shown in Additional file 1: Fig. S10a). In this third phase of the simulations, we allowed the Langevin dynamics to proceed for another 10^5^ iterations with these additional bond potentials turned on, which was sufficient to bring loop anchors in contact and equilibrate the looped structures. This process led to predicted contact frequency matrices that display sub-megabase block-like domains of enriched contacts along the diagonal, similar to TADs in experimental Hi-C data (Fig. 4b, right and Additional file 1: Fig. S10c). Energy potentials for A/B compartments were kept active during all phases of the simulation. We run 102 independent simulations starting from phase 1, and each of the 102 configurations obtained at the end of phase 2 was used as initialization to introduce variable numbers of loops, resulting in a total of 102 × 8 = 816 simulated configurations (Fig. 4). Predicted contact frequency matrices as shown in Fig. 4b were obtained by averaging over 100 independent configurations and display both A/B compartments and TADs. All simulations were performed using the LAMMPS molecular dynamics simulation framework (https://lammps.sandia.gov/) [65].

### Generation of simulated images of chromosome regions

We used the predicted 3D polymer configurations for chromosome 1 to generate simulated 3D super-resolution images of chromosome regions for different loop densities, as illustrated in Additional file 1: Fig. S11a. For each loop density, we split each of the 102 simulated polymer chains of 249 Mb into 349 sub-chains to simulate SCE, according to the expected probability density after 6 rounds of cell division (Additional file 1: Fig. S2b). Very small chains (less than 5 Mb) are excluded, but the resulting distribution is still consistent with the theoretical prediction (Additional file 1: Fig. S2d). Next, we distributed 600 localizations per megabase along the sub-chains, using linear interpolation to determine positions between beads and assuming a uniformly random probability density along the genome. To simulate background noise, we added uniformly random localizations in a cubic volume surrounding each sub-chain, with a density of 150 localizations per μm^3^ as determined from the average background of experimental data. Finally, these localizations were randomly displaced using a normal distribution of standard deviation α = 65 nm, chosen in order to match the Fourier ring correlation [47] estimation of the resolution of our experimental data (Additional file 1: Fig. S11b, S4e).

### Resources

#### Cell lines

HCT-116 cells used for experiments in Figs. 1 and 2, Additional file 1: Fig. S1, S5 are from ATTC and were authenticated by ATCC. For cohesin degradation experiments (Fig. 3), we used HCT-116-RAD21-mAID-mClover cells (HCT-116-RAD21-mAC) kindly provided by Prof. Kanemaki (National Institute of Genetics, Japan).

#### Software

Super-resolution images were reconstructed with the open access ImageJ plugin ZOLA-3D (version 0.2.4). Molecular dynamics simulations were performed using the open access software LAMMPS (version: lammps/17Nov16). Scripts to define the initial configurations of the polymers were written in Python 3.8. The number of imaged and simulated chromosome regions is summarized in Additional file 1: Table 1.

## Supplementary Information

**Additional file 1:** Figures S1-S16 and Table S1.

**Additional file 2:** Video S1. 3D visualization of a chromosome in G1 phase with super-resolution (left) and the corresponding widefield image (right).

**Additional file 3:** Video S2. 3D visualization of five chromosomes in G1 phase (top) and five chromosomes in M phase (bottom).

**Additional file 4:** Video S3. 3D visualization of five chromosomes without auxin (top) and five chromosomes with auxin treatment (bottom). The second and fourth rows show the same images after segmentation and background removal.

**Additional file 5:** Video S4. 3D visualization of five simulated chromosomes with 25 loops/Mb (top) and without loops (bottom). The second and fourth rows show images obtained after adding random localization errors and background noise. The exact same chromosome region is shown within each column.

**Additional file 6:** Review history.

## Data Availability

The datasets used and/or analyzed during the current study as well as the code for image analysis are available on Github [66]. The code is also available on Zenodo [67]. To define A/B compartments we used Hi-C data from [8] with accession number GSE104333. To analyze AT/GC content, we used human genome GC percent data downloaded from the UCSC website [68].
