# Supplementary material for: Super-resolution visualization and modeling of human chromosomal regions reveals cohesin-dependent loop structures
Source: Genome Biol. 2021 May 11;22:150. doi: 10.1186/s13059-021-02343-w (PMC8111965; doi:10.1186/s13059-021-02343-w)
Supplement: Supplementary file 1 — Additional file 1: Figures S1-S16 and Table S1. [file 13059_2021_2343_MOESM1_ESM.docx]

**Additional file 1**

**Figures S1-S16 and Table S1**

**Super-resolution visualization and modeling of human chromosomal regions reveals cohesin-dependent loop structures**

Xian Hao^1,2^, Jyotsana J Parmar^1,4^, Benoît Lelandais^1^, Andrey Aristov^1^, Wei Ouyang^1,3^, Christian Weber^1^, Christophe Zimmer^1^

^1^Institut Pasteur, Imaging and Modeling Unit; UMR 3691, CNRS; Paris, France

^2^School of Public Health & Jiangxi Provincial Key Laboratory of Preventive Medicine, Nanchang University, Nanchang 330006, China

^3^Université de Paris, F-75013, Paris, France

^4^Simons Center for the Study of Living Machines, National Center for Biological Sciences (TIFR), Bangalore 560065, Karnataka, India

[**Fig. S1: Assessing sister chromatid exchange with metaphase spreads**](#_n8ngr4nei35k) **2**

[**Fig. S2: Predicted genomic sizes of imaged chromosome regions**](#_jeg2hfc0v7il) **3**

[**Fig. S3: ZOLA-3D super-resolution imaging method**](#_opl8zgs1i14f) **4**

[**Fig. S4: Estimated resolution of imaged chromatin regions**](#_rzuqdjg03kyb) **5**

[**Fig. S5: Super-resolution images of chromosomes in G1 vs M phase**](#_yz1rwb25unpq) **6**

[**Fig. S6: Analysis of super-resolution images of chromatin**](#_1gwyxn6l826y) **7**

[**Fig. S7: Distributions of structure parameters in experiments and simulations**](#_ly75meurp5rm) **8**

[**Fig. S8: Statistical comparisons of gyration radii and smoothness**](#_7krxldfjr8aa) **9**

[**Fig. S9: Simulating A/B compartments**](#_mvwfv4yn1t9m) **11**

[**Fig. S10 : Simulating random distributions of loops**](#_i123dralpk7q) **12**

[**Fig. S11: Simulating 3D super-resolution images of chromosome regions**](#_31fscsio5zrc) **13**

[**Fig. S12: Predicted high density regions arise from A/B compartmentalization**](#_6j5t95dc1r09) **15**

[**Fig. S13: Quantifying chromatin structures for different simulation parameters**](#_51v4vrra49xl) **16**

[**Fig. S14: Simulations predict apparent contact domains in single cells**](#_5l0ei6q40c5v) **17**

[**Fig. S15: Effect of non-uniform AT content on localization density**](#_mm67eu8fzt7j) **18**

[**Fig. S16: Combining Oligo-FISH with EdU-based staining**](#_6e2t8yovnja) **20**

[**Table S1: Number of imaged and simulated chromosome regions**](#_phk94fh2amh2) **21**

[**Supplementary References:**](#_5pfe7tdtbpxh) **22**

# Fig. S1: Assessing sister chromatid exchange with metaphase spreads

**a**) Microscopy images of metaphase spreads, with DAPI staining in green and F-ara-EdU in red for different concentrations of F-ara-EdU (5 µM, 30 µM, 100 µM). Sister chromatid exchange (SCE) leads to incomplete EdU labeling of one chromatid and a partial labeling of its sister chromatid, as shown in insets. **b**) Percentage of chromosomes exhibiting SCE as a function of F-ara-EdU concentration. Data shown are mean +/- standard deviation. The red dashed line is a linear fit for F-ara-EdU concentrations ranging from 1 µM to 50 µM. The extrapolated frequency for a F-ara-EdU concentration of zero is 14.4%. At the concentration of 10 µM used for all super-resolution imaging experiments, the SCE percentage is 18%. This corresponds to 1.37 SCE events per Gb of DNA, on average.


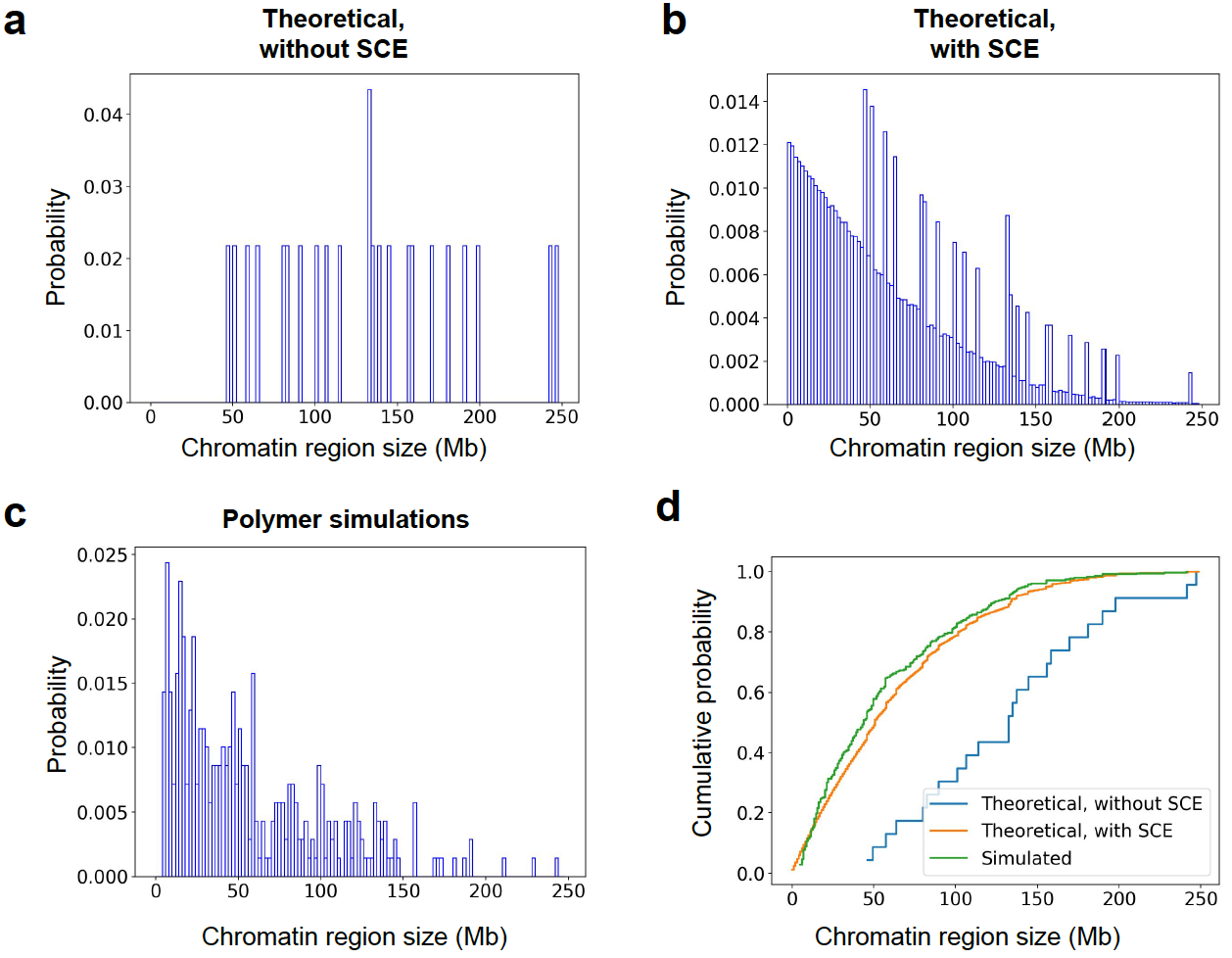


# Fig. S2: Predicted genomic sizes of imaged chromosome regions

**a**) Distribution of the genomic sizes of chromosomes in the human genome. **b**) Predicted distribution of the genomic size of imaged chromatin regions after six rounds of cell division, assuming that SCE events obey Poissonian statistics with a rate of 1.37 SCE per Gb of DNA per cell division (see **Fig. S1b**). The peaks correspond to the sizes of intact chromosomes, not affected by SCE. **c**) Actual distribution of imaged chromatin regions in polymer simulations. 102 simulated chromosomes of 250 Mb each were split into 349 sub-chains to simulate SCE and match the probability distribution of **b** (see **Fig. S11**). **d**) Cumulative probability distributions of **a** (blue), **b** (orange) and **c** (green).

# Fig. S3: ZOLA-3D super-resolution imaging method

Overview of the ZOLA-3D imaging approach [[1]](https://www.zotero.org/google-docs/?eTAwqi). **a**) ZOLA-3D uses a deformable mirror to generate point spread functions (PSFs) suitable for 3D localization of single molecules from 2D images. We used a saddle point PSF for all super-resolution imaging experiments. Z-stacks of fluorescent beads are acquired over an axial range of 4 μm, with Δz =50 nm (top). ZOLA-3D uses these images to compute a maximum likelihood estimation (MLE) of the phase (middle). A realistic model of the PSF is computed from the retrieved phase (bottom). **b**) 3D super-resolution image reconstruction with ZOLA-3D. 80,000 frames of single molecules are acquired (top). 3D localizations of single molecules are then computed by MLE fitting of the calibrated PSF model to these images. Redundant 3D cross-correlation is used to correct 3D spatial drift. Filtering ensures that remaining localizations have a goodness-of-fit ($\chi^{2}$) below 2, as well as predicted lateral and axial localization precisions (Cramer-Rao lower bounds) $\sigma\leq$30 nm and 60 nm, respectively (see **Fig. S4**). Localizations that are detected in close proximity to each other (distance <140 nm) in consecutive frames are merged. The rendered image (bottom right) shows an example of an EdU-labeled chromatin region over an axial range of 3 µm, displayed as in **Fig. 2b**.

#

# Fig. S4: Estimated resolution of imaged chromatin regions

**a-d**) Histograms show the distributions of estimated localization precisions (Cramer-Rao lower bounds) along the x (**a**), y (**b**) and z (**c**) coordinates, and goodness-of-fit ($\chi^{2}$) (**d**), as computed from the images of chromatin regions in the modified HCT-116 cells (HCT-116-RAD21-mAC) with (n=43) and without (n=50) auxin treatment (**Fig. 3**). Solid curves show averages over the 43 or 50 chromatin regions, shaded areas show standard deviations. (**e**) Violin plots indicate the lateral resolutions estimated by Fourier Ring Correlation (FRC) [[2]](https://www.zotero.org/google-docs/?Jp1ILf) for the same two sets of images. The FRC provides a conservative resolution estimate that is affected by localization precision, sampling and residual drift. (**f**) Cumulative distribution functions of the gyration radii $R_{g, x}^{2}=\frac{1}{N}\sum_{i=1}^{N} {\|x_{i}-\frac{1}{N}\sum_{j=1}^{N} x_{j}\|}^{2}$, $R_{g,y}^{2}=\frac{1}{N}\sum_{i=1}^{N} {\|y-\frac{1}{N}\sum_{j=1}^{N} y_{j}\|}^{2}$and $R_{g,z}^{2}=\frac{1}{N}\sum_{i=1}^{N} {\|z_{i}-\frac{1}{N}\sum_{j=1}^{N} z_{j}\|}^{2}$along x, y and z, showing that imaged chromosome regions have smaller axial than lateral extent and are within the 3 µm axial range captured by ZOLA-3D (see **Fig. S3**).

# Fig. S5: Super-resolution images of chromosomes in G1 vs M phase

**a,b**) Example images of HCT-116 chromosomes in G1 phase (**a**) and M phase (**b**), displayed as in **Fig. 2b**. For animated 3D views, see Additional file 3: **Video S2**. **c-e**) Violin plots compare the distributions of localization counts, gyration radii and smoothness between chromosomes in G1 phase (n=34) and M phase (n=27). Differences are highly significant (two-sided rank-sum test, ** p<10^-2^; **** p<10^-4^). **f,g**) Gyration radii and smoothness as a function of localization counts . The gyration radius correlates strongly with the number of localizations, whereas smoothness does not (Spearman correlation coefficients *r* and associated p-values are indicated).

#

#

#

# Fig. S6: Analysis of super-resolution images of chromatin

**a**) An example super-resolution image reconstructed with ZOLA-3D [1]. A region of interest (ROI) is manually selected in the background (dashed rectangle). **b**) A Voronoi-based segmentation of the image is then performed by automatically selecting localizations whose local density (at rank 2) is 4 times higher than the average local density in the background [5]. **c,d**) Concave hulls of the segmented image for two levels of spatial coarse-graining: fine (**c**) and coarse (**d**). The concave hulls are obtained by selecting tetrahedra whose circum-ellipsoids have lateral radii smaller than 𝞪=𝞪_1_=100 nm (**c**) or 𝞪= 𝞪_2_=500 nm (**d**) (and axial radii smaller than 200 nm and 1 µm, respectively). The smoothness $S$ is defined as the ratio between the volumes of these concave hulls: $S=V_{\alpha_{1}} / V_{\alpha_{2}}$. See “Methods” for details. **e,f**) Gyration radius (**e**) and smoothness (**f**) as function of genomic size for n=349 x 6 images of simulated chromosome regions (see **Fig. S11**). Gyration radius correlates strongly with chromosome region size whereas smoothness does not (Spearman correlations *r* and p-values as indicated).


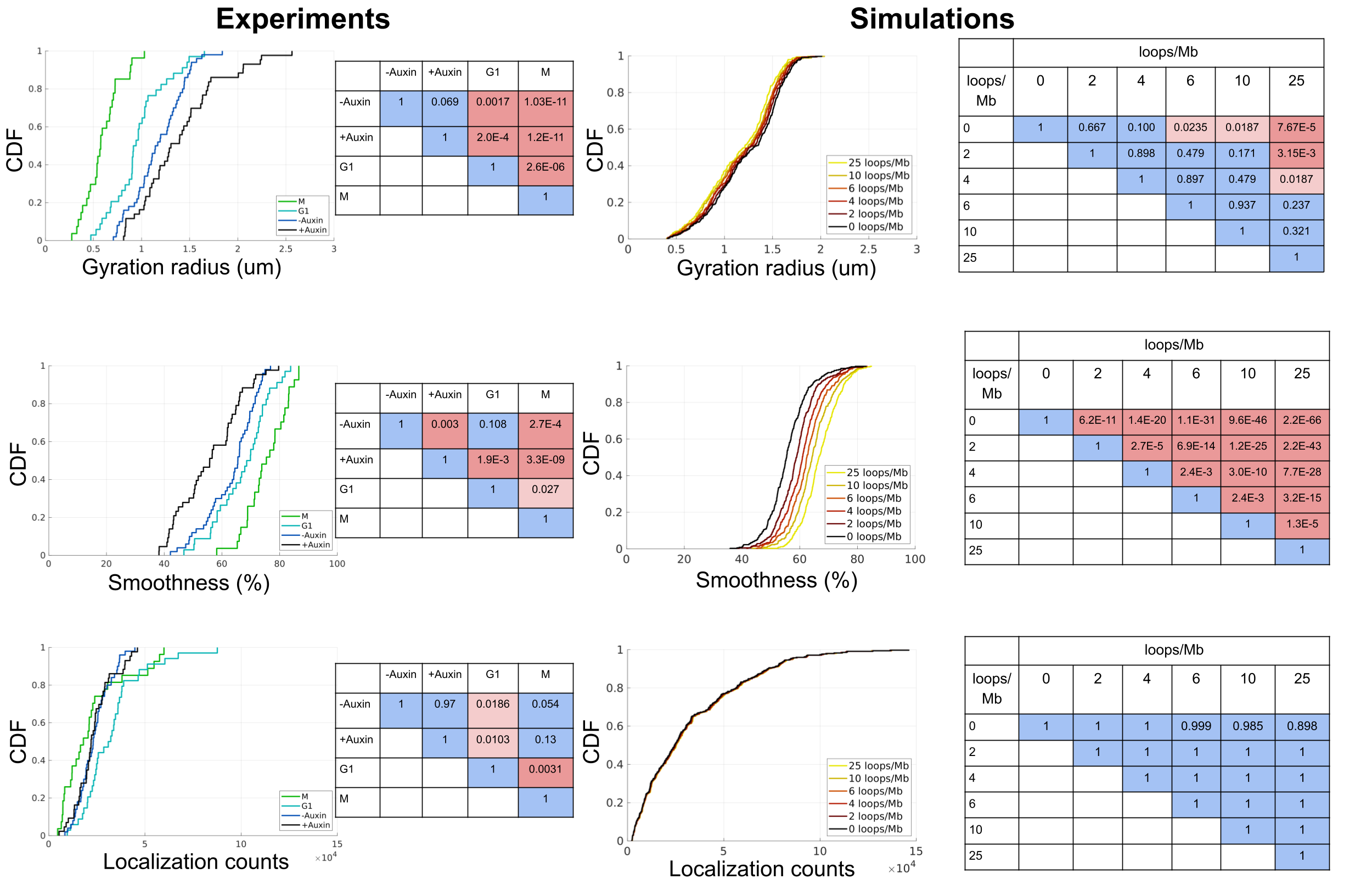


#

# Fig. S7: Distributions of structure parameters in experiments and simulations

Cumulative distribution functions (CDFs) of gyration radii (top), smoothness (middle) and localization counts (bottom) in experimental data (left) and simulations (right). The experimental imaging data are from chromosomes in wild-type HCT-116 cells imaged in M phase (green) or G1 phase (cyan) (**Fig. S5**), and modified HCT-116 cells (HCT-116-RAD21-mAC) [[3]](https://www.zotero.org/google-docs/?VeTpFh) imaged in presence of auxin (black) or left untreated (dark blue). The simulation data are from models with different numbers of loops from 0 (black) to 25 loops/Mb (yellow). The tables indicate the results (p-values) of Kolmogorov-Smirnov tests between all pairs of distributions. Blue indicates non-significant differences (p>0.05), light pink indicates intermediate significance (0.01<p<0.05), dark pink indicates highly significant differences (p<0.01).

# Fig. S8: Statistical comparisons of gyration radii and smoothness

Legend see next page

**Fig. S8: Statistical comparisons of gyration radii and smoothness**

**a,b**) Violin plots compare the distribution of gyration radii (**a**) and smoothness (**b**) between imaged chromosome regions of wild-type HCT-116 cells (“WT”) and genetically modified HCT-116 cells (HCT-116-RAD21-mAC) in absence of auxin treatment (“Mut, -auxin”) [[3]](https://www.zotero.org/google-docs/?aqYWjL). The measured gyration radii differ significantly (p=0.0014). Differences in smoothness are only marginally significant (p=0.0485) (two-sided rank-sum test). **c-f**) Bootstrap analyses to determine the robustness of statistical differences in gyration radii (left) or smoothness (right) between different experiments *A* and *B*. For each parameter (e.g. gyration radius), we randomly select a subset of *n_A_* samples out of the total of *N_A_* samples from experiment *A* and a random subset of *n_B_* samples out of the total of *N_B_* samples from experiment *B* and compare the medians of the two subsets using a two-sided rank sum test. This is repeated 10,000 times. Histograms show the resulting distributions of *p*-values. Percentages in red, pink and violet indicate the percentage of *p*-values that are highly significant (p<0.01, darker pink shade), moderately significant (0.01<p<0.05, light pink shade) and not significant (p>0.05, blue shade), respectively. **c**) Comparison of wild-type HCT-116 cells (“WT”) and modified HCT-116 cells in absence of auxin treatment (“Mut, -auxin”) [[3]](https://www.zotero.org/google-docs/?V4p7hm). Here, *n_A_*=30, *N_A_*=34 for “WT” and *n_B_*=45, *N_B_*=50 for “Mut, -auxin”. For gyration radii, differences are significant (p<0.05) in 100% of tests and highly significant (p<0.01) in >92% of tests. The reason for this difference is unclear. For smoothness, the differences are not significant (p>0.05) for 60% of tests, suggesting that the smoothness distributions are in fact similar. **d**) Comparison of modified cells treated with auxin (“+auxin”) or left untreated (“-auxin”). Here, *n_A_* =38, *N_A_*=43 for +auxin and *n_B_* =45, *N_A_*=50 for -auxin. Differences in gyration radii are significant (p<0.05) in 89% of tests. Differences in smoothness are significant (p<0.05) in 100% of tests and highly significant (p<0.01) in >99% of tests. This indicates that the reported differences in chromatin structure in presence vs. absence of auxin are robust to sampling. **e,f**) Intra-group controls, comparing structure parameters from auxin treated vs. auxin treated (**e,** *n_A_*=25, *N_A_*=50) and untreated vs. untreated cells (**f,** *n_B_*=22, *N_B_*=43). Intra-group differences are not significant (p>0.05) for >95% of tests, as expected.

#

#

# Fig. S9: Simulating A/B compartments

This figure compares Pearson correlation matrices of contact frequencies predicted by the polymer simulation in phase 3 (bottom left triangle) to their counterpart in Hi-C data [[4]](https://www.zotero.org/google-docs/?liNIbq) (top right triangle), for chromosomes 1 through 23 (from top left to bottom right). The genomic resolution (bin size) is 1 Megabase. The ‘plaid pattern’ characteristic of A/B compartmentalization apparent in the Hi-C data is approximately reproduced by our model.

#

# Fig. S10 : Simulating random distributions of loops

**a**) Schematic showing the positions of loops (black arcs) along a 5 Mb segment of chromosome 1 for different assumed loop densities, from zero (no loops, top) to 25 loops per Mb (bottom). The positions of loop anchors are defined by a process that simulates random landing of an extruding complex (e.g. cohesin) on the DNA followed by bidirectional extrusion [[5,6]](https://www.zotero.org/google-docs/?TwOfmN) with an average processivity of 250 Kb. Red dots indicate the positions of obstacles to loop extrusion (CTCF bound sites). Loops are allowed to form within other loops, whose anchors then act as additional obstacles to extrusion. The average number of loops per Mb and the corresponding percentage of DNA enclosed by loops are indicated on the left and right, respectively. **b**) Histogram shows the distribution of intervals between CTCF sites (red dots in **a**) drawn from the probability density $f(L) = L/L_{0}e^{-L/L_{0}}$ where $L_{0}$ was set to 165 Kb. The average interval is 320 Kb (median 275 Kb). See “Methods” for details. **c**) Contact matrix of a 5 Mb segment of chromosome 1 as predicted by the simulation with 15 loops/Mb (25 Kb bins, average from 200 configurations, logarithmic scale). Color bar shows natural logarithm of contact counts.

#

#

# Fig. S11: Simulating 3D super-resolution images of chromosome regions

Legend see next page

**Fig. S11: Simulating 3D super-resolution images of chromosome regions**

This figure illustrates our approach for generating 3D super-resolution images of chromosomes based on our polymer simulations. **a,b**) From polymer models to realistic 3D super-resolution images. **a**) We start from 102 distinct 3D configurations of a polymer model for chromosome 1 (first column from left) as obtained directly from our molecular dynamics simulation [[7]](https://www.zotero.org/google-docs/?RHwMm3) (**Fig. 4**). We then simulate the effect of sister chromatid exchange (SCE) after 6 rounds of cell division by splitting the polymer at random locations (with probabilities as estimated in **Fig. S1b**), resulting in 349 distinct 3D models of chromosome regions (second column from left). Chains of less than 5 Mb are discarded, but the resulting distribution of polymer sizes is still consistent with the theoretical prediction (**Fig. S2d**). Finally, we create artificial 3D single molecule localization microscopy images by randomly sampling a finite number of localizations from the 3D models, and adding random background noise and random localization errors. Specifically, we used 600 localizations per Mb, added 150 random background localizations per µm^3^ and Gaussian distributed random localization errors with a standard deviation $\sigma=$65 nm. These parameters were chosen based on the experimental data to match the FRC resolution [[2]](https://www.zotero.org/google-docs/?Gm5Mxv) measured in the images (compare panel **b** to **Fig. S4e**). **c**) Simulated 3D images of chromatin regions with varying numbers of loops. The genomic size of the modeled region ranges from 15 to 173 Mb, as indicated. The assumed loop density increases from zero (top) to 25 loops per Mb (bottom). Seven independently simulated configurations are shown for each loop density, with color coding of the axial coordinates as in **Fig. 2a**. These images were generated assuming 600 localizations per Mb and no localization errors ($\sigma=0$). See Additional file 5: **Video S4** for animated 3D views with and without localization errors.

#

#

# Fig. S12: Predicted high density regions arise from A/B compartmentalization

**a,b**) Images predicted by our polymer simulations with or without A/B compartments. Simulations with A/B compartments (see **Fig. S9,** and “Methods”) predict the appearance of high density regions (**b**) that are not observed in images generated from simulations without A/B compartments (**a**). This is true irrespective of the inclusion of chromatin loops (compare images for 0 vs 25 loops/Mb). Images are simulated as described in **Fig. S11**.

#

# Fig. S13: Quantifying chromatin structures for different simulation parameters

Violin plots show the distributions of gyration radii and smoothness parameters as function of chromatin loop density as in **Fig. 5c,d,** but for different simulation parameters. The distribution of localization counts is also shown. **a**) Simulation parameters as described in the main text and “Methods”. **b-d**) Polymers are simulated as in **a**, but images are simulated differently: using 1,200 instead of 600 localizations per Mb (**b**), or 300 instead of 600 localizations per Mb (**c**), or with localization errors of standard deviation 𝝈=15 nm instead of 𝝈=65 nm (**d**).

#

#

# Fig. S14: Simulations predict apparent contact domains in single cells

This figure shows simulated chromatin configurations (**a**,**e**), distance matrices (**b,c,f,g**) and contact frequency matrices within a 2.5 Mb chromatin region as predicted by our polymer model without loops (**a**-**d**) or with 30 loops per Mb (**e-h**). The three distance matrices in **b** and **f** each correspond to a single simulated polymer configuration (shown in **a** and **e**, respectively, where each dot is the center of mass of a 25 Kb region). The distance matrices in **c** and **g** are averages over 100 independent configurations for the same 2.5 Mb region. Color shows the distance (in nm) between two beads averaged over 25 Kb, as indicated by the color bars. Note that block-like domains of small distances (red), reminiscent of TAD-like structures, are often visible in the distance maps of single chromosome configurations, both with (**f**) and without (**b**) loops. In absence of loops, the averaged distance map displays no structure (**c**), in agreement with the contact frequency matrix (**d**). In presence of loops, the averaged distance map (**g**) shows block-like domains of smaller distances on the diagonal that agree with the predicted contact domains (**h**).

#

# Fig. S15: Effect of non-uniform AT content on localization density

In this figure, we analyze the effect of non-uniform AT content on our imaging technique and define the conditions under which labeling can be considered as uniformly random, or unbiased. Because the EdU based imaging approach relies on incorporation of modified thymidines into replicating DNA, the labeling density is in principle biased by AT/GC content, potentially complicating the interpretation of imaged chromatin structures. An extreme example are chromatin regions with 100% GC content, which will be entirely invisible. More generally, differences in localization densities within a chromosome could potentially reflect differences in AT/GC content between different chromatin regions rather than differences in chromatin density. However, localization densities along a chromosome will vary even for a uniform AT/GC content and constant chromatin density, simply because incorporation of modified thymidines instead of native thymidines is incomplete and random. We now estimate and compare these two sources of variability -non-uniform AT/GC content and random labeling- and consider labeling to be unbiased if the latter variability dominates over the former.

To address this, we consider an imaged chromosome region partitioned into $m$ consecutive segments of equal length $l$ (number of base pairs). We define $c_{k}\in\left[ 0,1 \right]$ as the fraction of AT in segment $\#k$, $(k=1..m)$ and $n_{k}$ as the number of EdU localizations originating from that segment. Because incorporation of labeled thymidines is stochastic, $n_{k}$ is a random variable that obeys a binomial distribution with mean $\bar{n_{k}}=p lc_{k}$, where $p$ is the probability that a modified thymidine replaces a native one and is localized once. We estimate $p\sim{10}^{-3}$ from our experimental data based on the median number of localizations (~25,000) and the predicted median chromatin region size (~50 Mb). For segment $\#k$, stochastic labeling therefore introduces a variability in localization counts $n_{k}$ with a variance: ${\sigma_{stoch, k}^{2}=p(1-p) l c}_{k}$, and the average over the entire chromosome region is $\sigma_{stoch}^{2}=\frac{1}{m}\sum_{k=1}^{m} \sigma_{stoch, k}^{2}=p(1-p) l \mu$, where $\mu$ is the average AT fraction over that region ($\mu=\frac{1}{m}\sum_{k=1}^{m} c_{k}$). On the other hand, the variability in mean localization counts caused by non-uniform AT content is given by the variance of $\bar{n_{k}}=p lc_{k}$ along the chromosome region, i.e. $\sigma_{AT}^{2}=p^{2} l^{2} \sigma_{c}^{2}$, where $\sigma_{c}^{2}=\frac{1}{m}\sum_{k=1}^{m} \left( c_{k}-\mu\right)^{2}$ is the variance of the AT fraction over that region. Labeling can be considered as unbiased if: $\sigma_{stoch}^{2}$>$\sigma_{AT}^{2}$, or equivalently, if ${c_{k}>c}_{0}=p\left( 1-p \right)^{-1} l \sigma_{c}^{2}$. Panels **a** and **b** plot these quantities for genomic resolutions (segment lengths) $l$ ranging from $l$=1 bp to $l$=1 Mb and show the conditions for uniformly random (unbiased) labeling vs. non-uniform (biased) labeling.

**a**) Variances $\sigma_{stoch}^{2}$ (blue) and $\sigma_{AT}^{2}$ (orange) as function of $l$ using $\mu=59\%$ and $\sigma_{c}^{2}$ calculated for the human genome. **b**) Blue curve shows the percentage of AT in the human genome ($\mu$, with error bars showing $\sigma_{c}$). Orange curve shows $c_{0}(l)$, the minimum % of AT required for approximately unbiased labeling. Thus, for the vast majority of the human genome, our EdU based labeling approach is unbiased for genomic resolutions of ~100 Kb or smaller (left of the dashed vertical line).

Note that this analysis does not factor in the stochastic blinking of fluorescent labels, which will further increase the stochastic variability and further reduce the impact of non-uniform AT content.


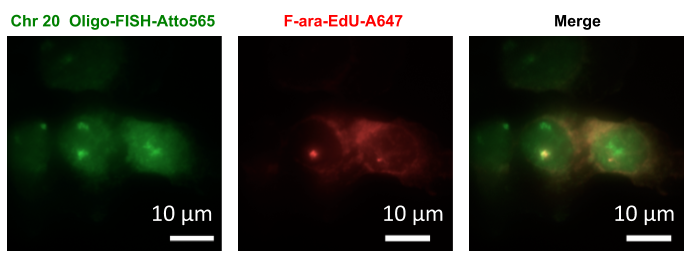


#

# Fig. S16: Combining Oligo-FISH with EdU-based staining

Widefield images of HCT116 cells with Oligo-FISH staining in green and F-ara-EdU staining in red. We used 92,000 Oligo-FISH probes conjugated to Atto565 dyes to target chromosome 20. F-ara-EdU was incorporated six generations before imaging using the dilution approach (**Fig. 1**) and click chemistry labeling with Alexa647 was performed after FISH. FISH with Oligopaint probes was performed following the protocols of [[8,9]](https://www.zotero.org/google-docs/?iKG2ZI).

#
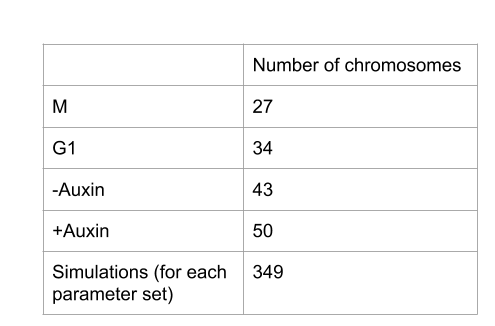


# Table S1: Number of imaged and simulated chromosome regions

This Table indicates the number of imaged chromosome regions for each of the four experimental conditions and the number of simulated chromosome regions. The indicated number of chromosomes approximately corresponds to the number of distinct cells imaged. For simulations with a given set of parameters (e.g. number of loops), we simulated 102 distinct chromosome configurations. Simulating the effect of sister chromatid exchange led to 349 distinct chromosome regions (see **Fig. S11**).

#

#

# Supplementary References:

[1. Aristov A, Lelandais B, Rensen E, Zimmer C. ZOLA-3D allows flexible 3D localization microscopy over an adjustable axial range. Nat Commun. 2018;9:2409.](https://www.zotero.org/google-docs/?h7eSdc)

[2. Banterle N, Bui KH, Lemke EA, Beck M. Fourier ring correlation as a resolution criterion for super-resolution microscopy. J Struct Biol. 2013;183:363–7.](https://www.zotero.org/google-docs/?h7eSdc)

[3. Natsume T, Kiyomitsu T, Saga Y, Kanemaki MT. Rapid Protein Depletion in Human Cells by Auxin-Inducible Degron Tagging with Short Homology Donors. Cell Rep. Cell Press; 2016;15:210–8.](https://www.zotero.org/google-docs/?h7eSdc)

[4. Rao SSP, Huang S-C, Glenn St Hilaire B, Engreitz JM, Perez EM, Kieffer-Kwon K-R, et al. Cohesin Loss Eliminates All Loop Domains. Cell. Cell Press; 2017;171:305-320.e24.](https://www.zotero.org/google-docs/?h7eSdc)

[5. Fudenberg G, Imakaev M, Lu C, Goloborodko A, Abdennur N, Mirny LA. Formation of Chromosomal Domains by Loop Extrusion. Cell Rep. Elsevier; 2016;15:2038–49.](https://www.zotero.org/google-docs/?h7eSdc)

[6. Sanborn AL, Rao SSP, Huang S-C, Durand NC, Huntley MH, Jewett AI, et al. Chromatin extrusion explains key features of loop and domain formation in wild-type and engineered genomes. Proc Natl Acad Sci. 2015;112:201518552.](https://www.zotero.org/google-docs/?h7eSdc)

[7. Arbona J-M, Herbert S, Fabre E, Zimmer C. Inferring the physical properties of yeast chromatin through Bayesian analysis of whole nucleus simulations. Genome Biol. 2017;18:81.](https://www.zotero.org/google-docs/?h7eSdc)

[8. Beliveau BJ, Apostolopoulos N, Wu C. Visualizing Genomes with Oligopaint FISH Probes. Curr Protoc Mol Biol. 2014;105:14.23.1-14.23.20.](https://www.zotero.org/google-docs/?h7eSdc)

[9. Beliveau BJ, Boettiger AN, Avendaño MS, Jungmann R, McCole RB, Joyce EF, et al. Single-molecule super-resolution imaging of chromosomes and in situ haplotype visualization using Oligopaint FISH probes. Nat Commun. Nature Publishing Group; 2015;6:7147.](https://www.zotero.org/google-docs/?h7eSdc)
