# Supplementary figures and images for: Super-resolution visualization and modeling of human chromosomal regions reveals cohesin-dependent loop structures
Source: Genome Biol. 2021 May 11;22:150. doi: 10.1186/s13059-021-02343-w (PMC8111965; doi:10.1186/s13059-021-02343-w)

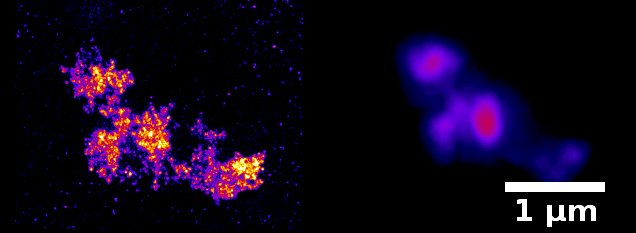

Supplement: Supplementary file 2 — Additional file 2: Video S1. 3D visualization of a chromosome in G1 phase with super-resolution (left) and the corresponding widefield image (right). [file 13059_2021_2343_MOESM2_ESM.gif]

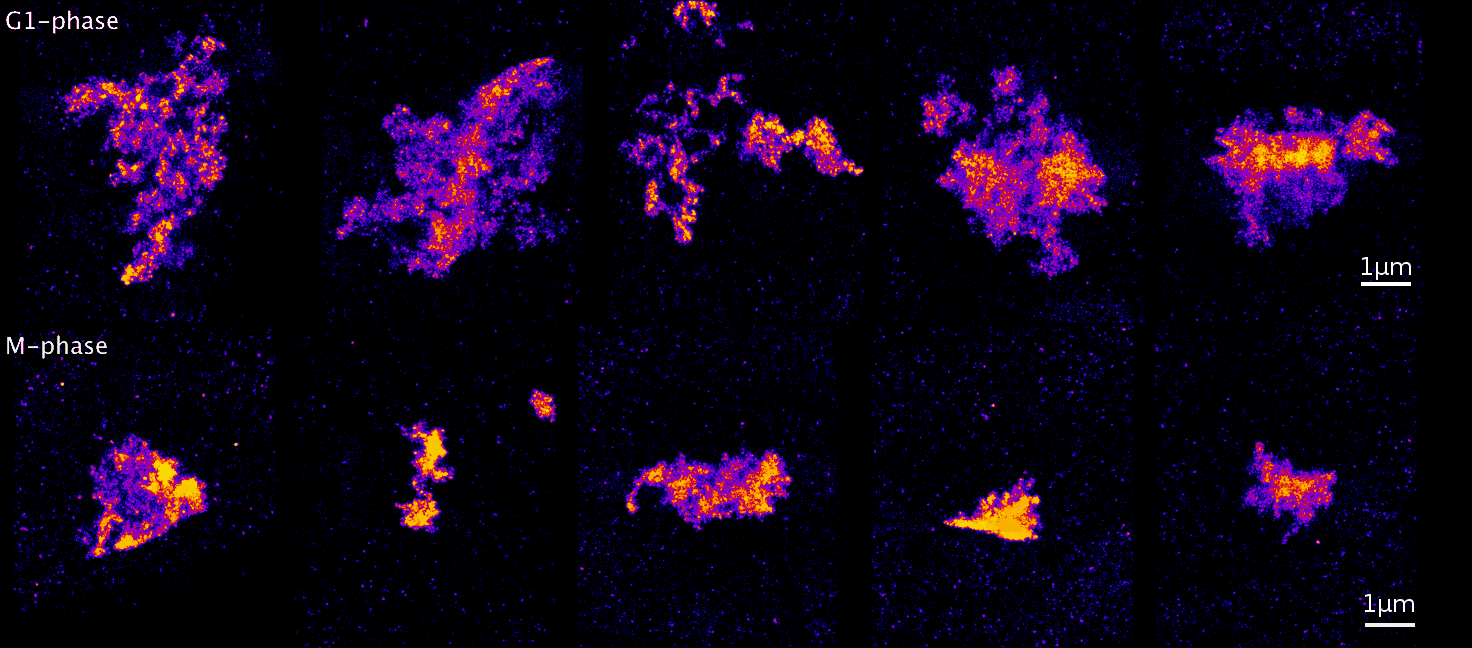

Supplement: Supplementary file 3 — Additional file 3: Video S2. 3D visualization of five chromosomes in G1 phase (top) and five chromosomes in M phase (bottom). [file 13059_2021_2343_MOESM3_ESM.gif]

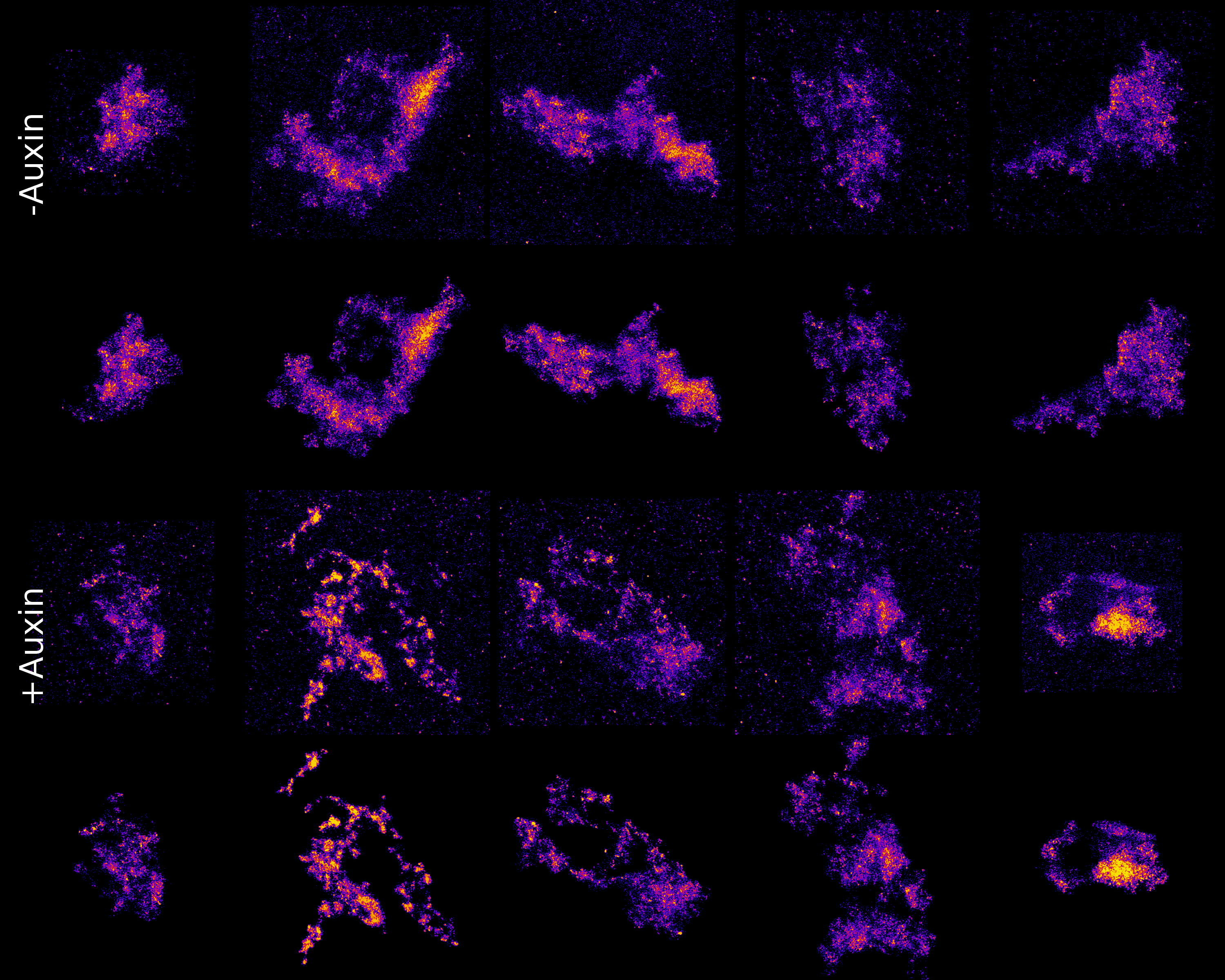

Supplement: Supplementary file 4 — Additional file 4: Video S3. 3D visualization of five chromosomes without auxin (top) and five chromosomes with auxin treatment (bottom). The second and fourth rows show the same images after segmentation and background removal. [file 13059_2021_2343_MOESM4_ESM.gif]

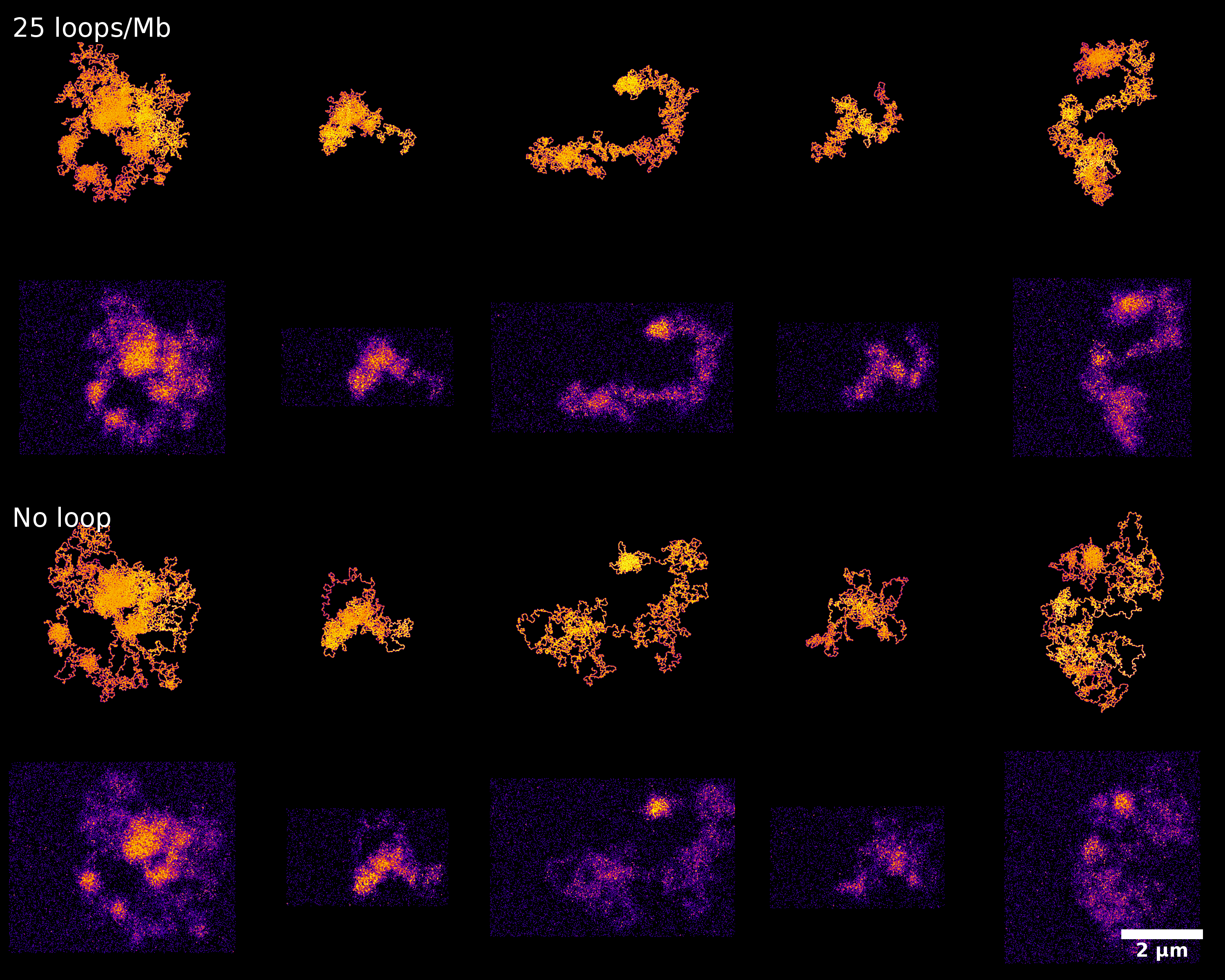

Supplement: Supplementary file 5 — Additional file 5: Video S4. 3D visualization of five simulated chromosomes with 25 loops/Mb (top) and without loops (bottom). The second and fourth rows show images obtained after adding random localization errors and background noise. The exact same chromosome region is shown within each column. [file 13059_2021_2343_MOESM5_ESM.gif]
