# Supplementary material for: Super-resolution visualization and modeling of human chromosomal regions reveals cohesin-dependent loop structures
Source: Genome Biol. 2021 May 11;22:150. doi: 10.1186/s13059-021-02343-w (PMC8111965; doi:10.1186/s13059-021-02343-w)
Supplement: Supplementary file 6 — Additional file 6: Review history. [file 13059_2021_2343_MOESM6_ESM.docx]

Review History

**First round of review**

**Reviewer 1**

**Are you able to assess all statistics in the manuscript, including the appropriateness of statistical tests used?** No, I do not feel adequately qualified to assess the statistics.

**Comments to author:**

Review of Super-resolution visualization and modeling of human chromosomal regions reveals cohesin-dependent loop structures

In this paper, Hao et al. report an elegantly simple method for visualizing whole and partial chromosome structures using sophisticated 3D SMLM: they label all chromosomes and then rely on dilution by division to eventually get single chromosome segments.

This is an elegant and powerfully simple method and a great complement to existing methods such as Hi-C and DNA-FISH (including derivates such as ORCA, oligoPAINT, etc.).

I think the primary impact of this paper is the development and validation of the method. Author do apply the method to study chromosome biology, and compare G1 and M chromosomes, chromosome structure with and without cohesin, and apply polymer modeling to estimate loop density.

Overall, I found the paper mostly convincing and since it introduces a very elegant, original and complementary method for chromosome structure determination, I think this paper could have a strong impact. I would support the publication of a revised manuscript addressing the comments below.

I think the authors could discuss some of the limitations that come from SCE a bit more explicitly. Please include an explicit discussion of limitation.

Page 8 "Because the frequency of loops in single cells is ill-determined from the Hi-C data," - Yes, while it is not very clear from Hi-C data, this has actually recently been estimated in two eLife papers: Cattoglio et al. 2019 and Holzmann et al. 2019. These papers found ~5 cohesins/Mb in mouse ES cells and ~20 cohesins/Mb in HeLa cells. Presumably, you'd get one loop for every one or two cohesins. The authors mention the Holzmann paper in the Discussion, but not Cattoglio which seems odd.

POLYMER MODELING: My understand is that the authors simulate cohesin-loops, but not compartmental interactions. This seems like a significant limitation. How would the authors result be affected if they modeled their chosen chromosome as simultaneously subject to cohesin loop extrusion and A/B-compartmentalization? For example, they could model their chromosome as a block co-polymer of A and B segments with preferential A-A and B-B interactions similar to Nubler 2018.

My concern is that the authors infer loop sizes from chromosome "compaction", gyration radius, sphericity etc. since natural chromosomes are subject to both A/B-compartmentalization and cohesin looping, it would seem to me that the authors might misestimate the cohesin loop density, if they do not also take A/B-compartmentalization into account.

What is the distribution of simulated polymers? Please compare the histogram to Fig. S2a?

Figure 5c: these plots are informative, but the changes in radius gyration and sphericity as a function of loop density is really small. How confident are the authors in their estimate? Could they give a confidence range? I found Figure S11 a little difficult to read.

Authors do most comparisons using median values. This seems reasonable, but could they also compare the full distributions in the supplement?

In Figure S1, the authors seem to say that the frequency of Sister Chromatid Exchange (SCE) depends on the F-ara-EdU concentration. Why would this be the case? I could not understand why SCE and concentration would be coupled?

Figure S2a is really important. I feel like the authors should show it or a simplified version (but not a box plot) in the main Figure 1, since this is crucial to understanding what the rest of the paper is imaging.

Page 3, Line 26: should also cite Gassler et al. EMBO 2017 for cohesin dependence.

Figure S3: typically, one would do a z-stack of a 100 nm TetraSpeck bead (such that the z-position is precisely known) and then test how well it fits with the inferred PSF model. This would be a required control to ensure the precision of the localization algorithm. Could the authors show a supplementary figure with such a control?

DATA:

I'd look to see a Supplementary Figure summarizing the imaging data, including:

How many cells/chromosomes were imaging?

How many SMLM localizations per imaged chromosome and a histogram of this for all imaged chromosomes?

I personally dislike box-plots and find them difficult to make sense of. This may just be a personal limitation, but could the authors show histograms, at least in the supplement (here referring to Figure 3c, please show the full histograms in the supplement also).

The authors can detect 3um axially. How did the authors ensure that the entire chromosome would be within their axial detection range?

Basically, for all calculated statistics (radius of gyration, sphericity, etc. ) could you please include summary statistics in the supplement and also histograms.

"Inspection of our experimental data also revealed large (up to ~3 μ m long) loose stretches in cells depleted of cohesin ( Figure 3e, arrows )." This is interesting, but could this mean the authors would be missing some of these in the experimental data due to the limited axial detection range?

The statement "The datasets used and/or analysed during the current study are available from the corresponding author on reasonable request." Seems a little inappropriate for modern science. Why not put the SMLM data on a public and free data repository?

**Reviewer 2**

**Are you able to assess all statistics in the manuscript, including the appropriateness of statistical tests used?** Yes, and I have assessed the statistics in my report.

**Comments to author:**

The manuscript 'Super-resolution visualisation and modeling of human chromosomal regions reveals cohesin-dependent loop structures' by Hao et al. provides a new angle on 3D genome architecture, in particular, looseness of chromatin loops which the group found to occur in great numbers and to be cohesin-dependent. This study is based on 3D imaging of nucleotide labeled nuclei in fixed human HCT116 cells using ZOLA-3D, an imaging and image analysis approach developed by the Zimmer group a few years ago, combined with whole genome coarse grain modeling. Although the methodologies have been used previously, they have not been combined to analyse large segments of the genome at high resolution. The modeling approach is original and does take advantage of an idea used to model the yeast genome by the Zimmer group which consists in varying the coarseness of a part of the genome (here the fragment to be analysed at 5 kb) compared to the rest of the genome to account for spatial constraints of all of the chromosomes within the nuclear space. The results shown are an important contribution to our 'C'- dominated view of genome organization, a view also based on models but models from contact frequencies and not actual folding patterns in situ.

The main conclusion put forward by the authors is the dependency of loops on cohesion based on degron depletion of Rad21 in a modified cell line. I find the claim too strong based on the evidence provided (see comments below). The work presented is a new piece to the puzzle and should be discussed as such.

The background section has to be improved. It is truly minimal, even shorter than the pre-submission inquiry itself. Please develop what is known about mitotic chromosomes. Make a logical transition to interphase chromosome organization.

Several imaging approaches, also at super-resolution and in live cells and not only using costly oligo-paint, developed in recent years enable analysis of genome organization in situ (Zidowska, Maeshima, Bystricky, Nollman) and so does electron-microscopy (for example Chrom-ID). In particular, Nozaki et al. 2018 described the role of cohesion in fiber folding.

At the end of the background (and after reading the abstract), the reader still does not know which cells were used in the study.

Major issues:

Please provide solid comparison between EdU labeled and unlabeled cells. Indeed, chromosome rearrangements via SCE were found to be frequent ('substantial'Figure S1). Is this also the case in unlabeled cells? Compare to the literature. What effect could EdU labeling, incorporation of labeled nucleotides have on fiber conformation, maintenance?

Please provide solid comparison between findings in the HCT116-RAD21-mAC and unmodified HCT116 cells. The modified cell line only expresses tagged RAD21 which may not be fully functional or may lead to aberrant structural features. This may not be visible using HiC as in the original paper (Rao et al. 2017) but could certainly have a local effect at the scale at which this study is operating.

Data shown suggest significant differences of loop parameters in wt and modified cells. Combine data from Figure 3f and S5c: the gyration radius is 0.94 µm in wt and 1.16 µm in -auxin RAD21-tagged cells. If the +auxin radius of 1.3 µm is a significant change compared to 1.16 µm, 0.94 µm relative 1.16 µm is as well. Sphericity is also significantly different: 9.94% in wt, 7.85% in -auxin vs 6.47% in +auxin modified cells. The main conclusion being based on the change in sphericity this has to properly assessed and discussed. The conclusions should be drawn from modeling also the wt images.

Additional questions:

The whole genome modeling approach is powerful. Would the deduced looping parameters change if the remainder of the genome was modeled at 1 Mb/5 Mb/ 10 Mb/ 100 Mb? Is so, how?

EdU labeling is random, but influenced by AT/GC content. Up to which % of AT labeling can be considered random? How does is influence results from telomeric regions for example?

Can EdU-labeling be combined with oligos? Is ZOLA-3D amenable for two-color image analysis?

Responses to these questions would be a definite plus to the article, but should at least be discussed.

Are the numbers of loops and folding parameters compatible with features of progressive genome decondensation and organization deduced from HiC in cell populations and single cells between M and G1 cell cyle phases (Dekker, Fraser/Tanay)?

**Authors Response**

**Point-by-point responses to the reviewers’ comments:**

Reviewer #1: Review of Super-resolution visualization and modeling of human chromosomal regions reveals

cohesin-dependent loop structures

In this paper, Hao et al. report an elegantly simple method for visualizing whole and partial chromosome structures using sophisticated 3D SMLM: they label all chromosomes and then rely on dilution by division to eventually get single chromosome segments.

This is an elegant and powerfully simple method and a great complement to existing methods such as Hi-C and DNA-FISH (including derivates such as ORCA, oligoPAINT, etc.).

I think the primary impact of this paper is the development and validation of the method. Author do apply the method to study chromosome biology, and compare G1 and M chromosomes, chromosome structure with and without cohesin, and apply polymer modeling to estimate loop density.

Overall, I found the paper mostly convincing and since it introduces a very elegant, original and complementary method for chromosome structure determination, I think this paper could have a strong impact. I would support the publication of a revised manuscript addressing the comments below.

**R1a) We thank the reviewer for his/her positive appreciation of the interest of our study.**

I think the authors could discuss some of the limitations that come from SCE a bit more explicitly. Please include an explicit discussion of limitationa.

**R1b) We agree that SCE merits more discussion and elaborate on SCE (and other limitations) in the Discussion. We also compare the estimated SCE frequency to the spontaneous SCE level. We find that our EdU concentration of 10 uM increases SCE frequency only moderately above background level. See the first subsection of the Results section and Figure S1. See also response R2e below.**

Page 8 "Because the frequency of loops in single cells is ill-determined from the Hi-C data," - Yes, while it is not very clear from Hi-C data, this has actually recently been estimated in two eLife papers: Cattoglio et al. 2019 and Holzmann et al. 2019. These papers found ~5 cohesins/Mb in mouse ES cells and ~20 cohesins/Mb in HeLa cells. Presumably, you'd get one loop for every one or two cohesins. The authors mention the Holzmann paper in the Discussion, but not Cattoglio which seems odd.

**R1c) Thank you for pointing this out. The Cattoglio et al. study should indeed have been cited here. We now mention both references in the relevant passage.**

POLYMER MODELING: My understand is that the authors simulate cohesin-loops, but not compartmental interactions. This seems like a significant limitation. How would the authors result be affected if they modeled their chosen chromosome as simultaneously subject to cohesin loop extrusion and A/B-compartmentalization? For example, they could model their chromosome as a block co-polymer of A and B segments with preferential A-A and B-B interactions similar to Nubler 2018.

My concern is that the authors infer loop sizes from chromosome "compaction", gyration radius, sphericity etc. since natural chromosomes are subject to both A/B-compartmentalization and cohesin looping, it would seem to me that the authors might misestimate the cohesin loop density, if they do not also take A/B-compartmentalization into account.

**R1d) We agree that it is interesting to revisit our analysis in the context of A/B compartments. We therefore followed the reviewer’s suggestion and extended our simulations to account for A/B compartments in addition to loops. Specifically, we partitioned the genome in A and B compartments based on the Hi-C data and introduced attractive energy potentials between B compartments. This led to predicted contact frequency correlation matrices in rather good agreement with Hi-C data (see new Figure S9).**

**Please note that in addition to including A/B compartments, we also made the simulations more realistic by changing the nuclear radius to 5.4 um, in better agreement with experimental observations (e.g. Cremer et al. PMID 33262376) than our earlier simulations (3.6 um).**

**Also, we revisited our estimate of resolution using a more conservative metric (see R1j below) and used this new estimate to create artificial images from the simulations, as explained in Figure S11.**

**Furthermore, we also improved our procedure for segmenting the localization data in a way that is more robust to background noise (see updated Methods) and by replacing our previous ‘sphericity’ metric by a ‘smoothness’ criterion, that is more robust to variations in chromatin region sizes, as shown by simulation predictions below (see also Fig S6e,f).**

**We computed predicted gyration radii and smoothness for different numbers of loops and compared them quantitatively to our imaging data. Our predicted gyration radii and smoothness values now agree even better with the experimental data than before: the predicted median gyration radius for simulations without loops is 1.30 um and the predicted median smoothness is 55.4%. This compares very well to the experimental measurements in cohesin depleted cells: 1.30 um and 55.8%, respectively. For simulations with large numbers of loops (25 loops/Mb) we predict gyration radii of 1.21 um and smoothness of 66.7%, which is very close to our experimental data in untreated cells: 1.16 um and 65.5%. Based on these new predictions, we estimate a minimum number of loops per Mb of 6-25, similar to our previous estimate (10 loops/Mb or more). We updated the Results and Discussion sections accordingly.**

What is the distribution of simulated polymers? Please compare the histogram to Fig. S2a?

**R1e) The genomic size distribution of simulated polymers follows the theoretical probability density derived from our estimate of SCE. To address the reviewer’s question we show the histogram of simulated chromatin region sizes in the new Figure S2c and compare the cumulative distribution functions directly in the new Figure S2d (green and orange curves).**

Figure 5c: these plots are informative, but the changes in radius gyration and sphericity as a function of loop density is really small. How confident are the authors in their estimate? Could they give a confidence range? I found Figure S11 a little difficult to read.

Authors do most comparisons using median values. This seems reasonable, but could they also compare the full distributions in the supplement?

**R1f) Compared to simulations without loops, adding 25 loops/Mb predicts a decrease in gyration radius of approximately 7% (from 1.30 to 1.21 um). This is indeed small, but the differences are highly significant, as indicated by our ranksum tests on medians (p=1E-4, Fig 5c). To address the reviewer’s comment, we show the full distributions (CDFs) and compare them using Kolmogorov-Smirnov (KS) tests in the new Fig S7. These tests confirm that the differences are significant. For example, comparing gyration radii for simulations with 25 loops/Mb to simulations without loops leads to p-values p<1E-4.**

**For smoothness (which replaces sphericity, see R1d above), the differences are larger and even more significant. For example, adding 25 loops/Mb predicts a smoothness increase of approximately 20% (from 55.4% to 66.7%) with p< 1E-69 from ranksum tests on the medians and p<1E-65 from KS tests on the distributions.**

**Our former Figure S11 (now part of Figure S8) is a bootstrap analysis to test whether statistical differences in gyration radii and smoothness with and without cohesin are robust. This is indeed the case, because comparing random subsets of the data almost always leads to statistically significant differences. We tried to better explain this analysis in our new version of this Figure.**

In Figure S1, the authors seem to say that the frequency of Sister Chromatid Exchange (SCE) depends on the F-ara-EdU concentration. Why would this be the case? I could not understand why SCE and concentration would be coupled?

**R1g) Yes, our data in Fig S1 indeed indicate that SCE is more frequent for higher concentrations of F-ara-EdU. We believe that the incorporation of modified nucleotides in EdU-labeling makes the DNA more fragile and increases the likelihood of DNA double strand breaks, which would also increase the likelihood of SCE.**

**We note that our observations are consistent with previous studies showing that SCE frequency increases with concentration of Bromodeoxyuridine (BUdR/BrDU), another thymidine analog (Heartlein et al. 1983- PMID: 6828032; Davidson et al. 1980 - PMID: 7354875, Wolff, 1977 - PMID: 74232).**

Figure S2a is really important. I feel like the authors should show it or a simplified version (but not a box plot) in the main Figure 1, since this is crucial to understanding what the rest of the paper is imaging.

**R1h) OK. In response to this comment, we expanded Figure 1 to illustrate the effect of SCE and include the histogram of predicted labeled chromatin region sizes (former Figure S2a).**

Page 3, Line 26: should also cite Gassler et al. EMBO 2017 for cohesin dependence.

**R1i) OK. We added this reference.**

Figure S3: typically, one would do a z-stack of a 100 nm TetraSpeck bead (such that the z-position is precisely known) and then test how well it fits with the inferred PSF model. This would be a required control to ensure the precision of the localization algorithm. Could the authors show a supplementary figure with such a control?

**R1j) We already determined the precision of our localization algorithm in our initial report of the ZOLA-3D method, using simulations, repeated imaging of fluorescent beads and of single molecules. These evaluations showed that our algorithm reaches the theoretical precision limit (Cramer-Rao bound). See Figures 1e,f and Supplementary Figures 5,6 in Aristov et al. Nat Comm, 8:2409 (2018). Therefore, the Cramer-Rao bounds plotted in Figure S4a-d can be taken as good estimates of the actual localization precision in the present study as well.**

**Please note however that resolution not only depends on localization precision, but also on the sampling density (ie on how many molecules are actually localized). In order to assess resolution more completely, we therefore added a new and much more conservative estimate based on Fourier Ring Correlation, which yields an estimated resolution of ~150 nm. See Figure S4e. We also took this new resolution into account when simulating images from our polymer model (see Figure S11 and Methods).**

DATA:

I'd look to see a Supplementary Figure summarizing the imaging data, including:

How many cells/chromosomes were imaging?

How many SMLM localizations per imaged chromosome and a histogram of this for all imaged chromosomes?

**R1k) OK. We included a new Table S1 that indicates the number of chromosomes/cells imaged in each condition (as well as the number of simulated chromosome regions). We also included CDFs of the number of localizations per chromosome region in the new Figure S7.**

I personally dislike box-plots and find them difficult to make sense of. This may just be a personal limitation, but could the authors show histograms, at least in the supplement (here referring to Figure 3c, please show the full histograms in the supplement also).

**R1l) We acknowledge that box plots are less informative than histograms, but visual comparison of histograms is less straightforward than for boxplots. To address the reviewer’s comment, we replaced the boxplots by violin plots, which show a smoothed version of the histograms but still facilitate visual comparisons. See new Figures 3, 5, S5, S8, S13.**

**In addition, we now show cumulative distribution functions in Fig. S7.**

The authors can detect 3um axially. How did the authors ensure that the entire chromosome would be within their axial detection range?

**R1m) Before acquiring super-resolution images of an EdU-labelled chromatin region, we checked that its axial extent was consistent with our ~3 um imaging range based on widefield image stacks. This led us to eliminate only very few cells, because the vast majority of EdU-labelled chromosome regions actually had an axial extent well below 3 um, even though the lateral extent was often larger, as shown by Figure S4f. This anisotropy reflects the fact that the nuclei of HCT116 cells are mostly flat, with an axial diameter roughly half the lateral diameter. We now mention this in the Methods section.**

Basically, for all calculated statistics (radius of gyration, sphericity, etc. ) could you please include summary statistics in the supplement and also histograms.

**R1n) We now include violin plots with medians and interquartile ranges in Figures 3 and 5, and in Figure S5) and cumulative distribution functions (CDFs) in Figure S7.**

"Inspection of our experimental data also revealed large (up to ~3 μ m long) loose stretches in cells depleted of cohesin ( Figure 3e, arrows )." This is interesting, but could this mean the authors would be missing some of these in the experimental data due to the limited axial detection range?

**R1o) In theory yes, but because of the small axial extent of EdU-labeled chromosome regions (see response R1m above), this is very rare.**

The statement "The datasets used and/or analysed during the current study are available from the

corresponding author on reasonable request." Seems a little inappropriate for modern science. Why not put the SMLM data on a public and free data repository?

**R1p) Absolutely. We uploaded our data (along with analysis code) on** [**https://github.com/imodpasteur/VoroQuant**](https://github.com/imodpasteur/VoroQuant) **and updated the relevant statement in the ‘Declarations’ section.**

Reviewer #2: The manuscript 'Super-resolution visualisation and modeling of human chromosomal regions reveals cohesin-dependent loop structures' by Hao et al. provides a new angle on 3D genome architecture, in particular, looseness of chromatin loops which the group found to occur in great numbers and to be cohesin-dependent. This study is based on 3D imaging of nucleotide labeled nuclei in fixed human HCT116 cells using ZOLA-3D, an imaging and image analysis approach developed by the Zimmer group a few years ago, combined with whole genome coarse grain modeling. Although the methodologies have been used previously, they have not been combined to analyse large segments of the genome at high resolution. The modeling approach is original and does take advantage of an idea used to model the yeast genome by the Zimmer group which consists in varying the coarseness of a part of the genome (here the fragment to be analysed at 5 kb) compared to the rest of the genome to account for

spatial constraints of all of the chromosomes within the nuclear space. The results shown are an important contribution to our 'C'- dominated view of genome organization, a view also based on models but models from contact frequencies and not actual folding patterns in situ.

**R2a) We thank the reviewer for appreciating our study’s contribution to the field and its complementarity to information provided by ‘C’-based techniques.**

The main conclusion put forward by the authors is the dependency of loops on cohesion based on degron depletion of Rad21 in a modified cell line. I find the claim too strong based on the evidence provided (see comments below). The work presented is a new piece to the puzzle and should be discussed as such.

**R2B) We agree with the reviewer that our work provides a piece of the puzzle, not necessarily the full picture. Our data are consistent with earlier claims by others, based mostly on population Hi-C and modeling, that interphase chromatin loops (or at least their vast majority) are dependent on cohesin and can be removed by degradation of a cohesin subunit. But we believe that our evidence that this view remains valid at the level of single cells is important, along with our independent estimates of chromatin loop frequency.**

The background section has to be improved. It is truly minimal, even shorter than the pre-submission inquiry itself. Please develop what is known about mitotic chromosomes. Make a logical transition to interphase chromosome organization.

Several imaging approaches, also at super-resolution and in live cells and not only using costly oligo-paint, developed in recent years enable analysis of genome organization in situ (Zidowska, Maeshima, Bystricky, Nollman) and so does electron-microscopy (for example Chrom-ID). In particular, Nozaki et al. 2018 described the role of cohesion in fiber folding.

**R2c) We had tried to be concise, but we acknowledge that our background section was perhaps too short and omitted previous studies that are worth mentioning. We have now expanded our background section to include these and related publications.**

**As for mitotic chromosomes: we first tried to elaborate on mitotic chromosomes, but then found that this interrupted the flow of the background section and distracted from the main focus of our study, which is on interphase chromosomes. We therefore chose not to dwell on mitotic chromosomes and instead to indicate more clearly in the background section and abstract that our focus is on interphase chromatin.**

At the end of the background (and after reading the abstract), the reader still does not know which cells were used in the study.

**R2d) We now mention at the end of the background and in the abstract that our study focuses on human cells.**

Major issues:

Please provide solid comparison between EdU labeled and unlabeled cells. Indeed, chromosome rearrangements via SCE were found to be frequent ('substantial'Figure S1). Is this also the case in unlabeled cells? Compare to the literature. What effect could EdU labeling, incorporation of labeled nucleotides have on fiber conformation, maintenance?

**R2e) We agree that the effect of EdU labeling is important to discuss in more detail.**

**Concerning sister chromatid exchange: Our metaphase spreads indeed revealed a significant amount of recombination, most likely caused by SCE. Our quantifications (Fig S1) indicate 18% SCE per chromosome, on average, for a (F-ara-)EdU concentration of 10 uM. For higher EdU concentrations, the measured SCE frequency increases approximately linearly up to 50 uM. This increase with concentration is consistent with previous literature on BrdU labeling, as mentioned above in response to reviewer 1 (response R1g), and we now indicate this in the revised text.**

**When we reduce EdU concentration from 10 uM to 1 uM, our measured SCE frequency is only reduced from 18 to 15%. Because detection of SCE requires both DAPI and EdU, we cannot directly assess SCE frequency in unlabeled cells. However, fitting a linear trend to concentrations from 1 to 50 uM predicts a SCE frequency of 14.5% in absence of EdU labeling (red dotted line in our updated Fig S1b). This number is in good agreement with prior estimates of spontaneous SCE in HCT-116 cells (see Kantidakis et al. 2016 - PMID 26883360, Figure 2A).**

**Therefore, although high concentrations of EdU indeed increase SCE substantially above spontaneous levels, the concentration chosen here (10 uM) only marginally increases SCE frequency above spontaneous levels (18% vs 14.4%, i.e. an 25% increase). We mention this in the revised text.**

**Concerning the effect of EdU labeling on fiber conformation and maintenance: we did not find any evidence for such effects in the literature, but we cannot rule out that incorporation of modified thymidines causes subtle changes in chromatin fiber conformation. However, we stress that unlike BrdU or EdU, F-ara-EdU labeling does not affect DNA replication or induce cell cycle arrest at the concentrations used for imaging here, as previously shown by Neef and Luedtke (PMID 22143759). Therefore, it appears unlikely that EdU labeling affects chromatin structure in any functionally meaningful way. Finally, we note that any potential effects of EdU labeling on chromatin structure would impact both auxin-treated and untreated cells, and therefore would not affect our interpretation of cohesin-dependent changes in chromatin structure. Nevertheless, we mention the theoretical possibility of labeling-induced changes in chromatin structure in the revised Discussion.**

Please provide solid comparison between findings in the HCT116-RAD21-mAC and unmodified HCT116 cells. The modified cell line only expresses tagged RAD21 which may not be fully functional or may lead to aberrant structural features. This may not be visible using HiC as in the original paper (Rao et al. 2017) but could certainly have a local effect at the scale at which this study is operating.

Data shown suggest significant differences of loop parameters in wt and modified cells. Combine data from Figure 3f and S5c: the gyration radius is 0.94 µm in wt and 1.16 µm in -auxin RAD21-tagged cells. If the +auxin radius of 1.3 µm is a significant change compared to 1.16 µm, 0.94 µm relative 1.16 µm is as well. Sphericity is also significantly different: 9.94% in wt, 7.85% in -auxin vs 6.47% in +auxin modified cells. The main conclusion being based on the change in sphericity this has to properly assessed and discussed. The conclusions should be drawn from modeling also the wt images.

**R2f) It is true that the gyration radii are significantly different in the WT G1 cells compared to the untreated modified cells, as indicated in the new Figure S8a (p=0.0014). We presently cannot provide a satisfying explanation for this difference in gyration radii. We cannot exclude that some technical change (eg in the cell culture conditions or imaging) is responsible for the differences in gyration radii. On the other hand we also cannot exclude a biological difference between the cell lines. However, if there is a biological difference, it is unclear to us what to expect and how to model it.**

**While the gyration radii differ significantly, please note that the differences in smoothness (which replaces sphericities, see R1d above) are only marginally significant according to our ranksum test (Figure S8b: p=0.0485) and are not significant according to a Kolmogorov Smirnov test (Figure S7, p=0.108) or to a bootstrap analysis (Figure S8c). Since smoothness is more robust to variations in the size of labeled chromatin regions (Figure S6e,f and R1d above) we consider it more important that these parameters are relatively similar in WT and modified cells than the difference in gyration radii. The absence of a clear difference in smoothness is consistent with the fact that chromatin morphologies are in fact similar in WT and modified cells (to the extent that can be captured by our technique).**

**At any rate, even if some biological or technical factor differs between WT and modified cells, our analysis of the effect of cohesin depletion only rests on a comparison of modified cells with and without auxin and not on a comparison with WT cells. Therefore, the main conclusions of our analysis of cohesin on loop structures still hold.**

Additional questions:

The whole genome modeling approach is powerful. Would the deduced looping parameters change if the remainder of the genome was modeled at 1 Mb/5 Mb/ 10 Mb/ 100 Mb? Is so, how?

**R2g) The short answer is yes, if we keep all other simulation parameters identical. Indeed, we repeated the simulations with a coarse graining of 2.5 Mb instead of 1 Mb, and in this case obtained the following predictions for gyration radii and smoothness parameters.**

**The predicted gyration radii for chromosome 1 are smaller, and the smoothness values are larger than in experimental data. The reason for this is that the other (coarse-grained) chromosomes expand faster when modeled with 2.5 times fewer beads, hence leaving less space for chromosome 1. This could potentially be remedied by letting the simulations equilibrate, but this would run against previous evidence by Rosa & Everaers, Mirny, Thirumalai and others that human chromosomes are in fact out of equilibrium (and require excessive computation time). Nevertheless we note that even if the predictions do not quantitatively agree with our experimental data, the qualitative behaviour is the same, i.e. increasing the number of loops in chromosome 1 still decreases predicted gyration radius and increases smoothness. Note also that a significant change in gyration radius still requires ~10 loops /Mb, thus supporting a similar estimation of looping frequency.**

EdU labeling is random, but influenced by AT/GC content. Up to which % of AT labeling can be considered random? How does is influence results from telomeric regions for example?

**The reviewer is right that AT/GC content can in theory influence labeling. In principle, strong variations of AT content along a chromosome could lead to strong variations in localization density. However in practice, the variations in localization density due to random incorporation and localization of EdU molecules are typically much larger than the variations due to inhomogeneities in AT content. We added a quantitative analysis of these two effects and an application for the human genome in a new Fig S15. When comparing distinct genomic regions inside a chromosome, the variations due to random EdU sampling overwhelm the effect of differences in AT % for regions up to 100 Kb in length (see Fig S15a). Equivalently, differences in AT % would have a notable effect at genomic resolutions of 100 Kb (respectively 10 Kb) only for AT percentages below ~30% (respectively 3.4%) (see Fig S15b). Such low levels of AT content are never reached in the human genome. Thus, non-uniform labeling is only an issue for unrealistically low AT percentages.**

**The AT content of vertebrate telomeres is 50% (since they are made up of repeated TTAGGG sequences), while human centromeres have AT content of ~65%, hence we do not expect any significant labeling biases with our approach for these regions.**

Can EdU-labeling be combined with oligos? Is ZOLA-3D amenable for two-color image analysis?

**Yes, EdU labeling can be combined with oligos. We added a new Supplementary Figure with dual color imaging of chromosomes with EdU and Oligopaint probes to illustrate this (Fig S16).**

**And yes, ZOLA-3D is amenable for dual color imaging. In fact, because ZOLA-3D uses reflective rather than refractive optics, it is less prone to chromatic aberrations than many other 3D SMLM systems. We illustrate this with dual color super-resolution imaging of microtubules below (alpha-tubulin proteins are labeled in red, beta-tubulin in green).**

Responses to these questions would be a definite plus to the article, but should at least be discussed.

Are the numbers of loops and folding parameters compatible with features of progressive genome decondensation and organization deduced from HiC in cell populations and single cells between M and G1 cell cycle phases (Dekker, Fraser/Tanay)?

**The Dekker/Mirny lab study by Gibcus et al (Science vol 359, issue 6376) used Hi-C and simulations to study the condensation of the chicken genome during mitosis and the role of condensin I and II. They propose that condensin II extrudes consecutive loops, with loop sizes increasing from 50 to ~800 Kb over time. Because loops are consecutive, this would imply a loop frequency decreasing from 21 to 1 loops /Mb. We note that 21 loops/Mb is close to our estimate of ~6-25 or more cohesin-dependent loops per Megabase in the interphase genome. We added a sentence to this effect in the Discussion.**

**In the study of the Fraser/Tanay labs by Nagano et al. (Nature 2017), single cell Hi-C was done on mouse ES cells during the cell cycle and 3D models were built, but their resolution (0.1, 0.5 and 1 Mb) is not sufficient to quantify cohesin dependent loops.**

**Second round of review**

**Reviewer 1**

The authors have fully addressed my comments, and I believe the revised manuscript is substantially strengthened and I fully recommend its publication. I would like to thank the authors for carefully and fully addressing my comments and answering my questions. I believe the method is an exciting addition to the arsenal of methods for studying chromosome conformation, and that it is likely to have a big impact.

**Reviewer 2**

The manuscript is truly improved. The authors extensively and satisfactorily addressed my concerns. Including some of the answers to my comments and criticisms in the text makes the article more comprehensive and the method more useful to the community. I also appreciate the suggestions by the other reviewer which were included in the revised manuscript as well. I highly recommend publication.

Minor comment: please delete 'very significantly', in particular, do not compare p values by saying significantly vs very significantly. For example on p 15. Simply state ",, increase of 12% (n=.., p=..) of .... and increase of 15% (n=.., p=..)...."the following sentence says '..small changes but statistical analysis/ bootstrap analysis show that these are significant.."
